# Translation initiation events on structured eukaryotic mRNAs generate gene expression noise

**DOI:** 10.1093/nar/gkx430

**Published:** 2017-05-17

**Authors:** Estelle Dacheux, Naglis Malys, Xiang Meng, Vinoy Ramachandran, Pedro Mendes, John EG McCarthy

**Affiliations:** 1 Warwick Integrative Synthetic Biology Centre (WISB) and School of Life Sciences, University of Warwick, Gibbet Hill, Coventry CV4 7AL, UK; 2 Center for Quantitative Medicine, UConn Health, 263 Farmington Avenue, CT 06030-6033, USA

## Abstract

Gene expression stochasticity plays a major role in biology, creating non-genetic cellular individuality and influencing multiple processes, including differentiation and stress responses. We have addressed the lack of knowledge about posttranscriptional contributions to noise by determining cell-to-cell variations in the abundance of mRNA and reporter protein in yeast. Two types of structural element, a stem–loop and a poly(G) motif, not only inhibit translation initiation when inserted into an mRNA 5΄ untranslated region, but also generate noise. The noise-enhancing effect of the stem–loop structure also remains operational when combined with an upstream open reading frame. This has broad significance, since these elements are known to modulate the expression of a diversity of eukaryotic genes. Our findings suggest a mechanism for posttranscriptional noise generation that will contribute to understanding of the generally poor correlation between protein-level stochasticity and transcriptional bursting. We propose that posttranscriptional stochasticity can be linked to cycles of folding/unfolding of a stem–loop structure, or to interconversion between higher-order structural conformations of a G-rich motif, and have created a correspondingly configured computational model that generates fits to the experimental data. Stochastic events occurring during the ribosomal scanning process can therefore feature alongside transcriptional bursting as a source of noise.

## INTRODUCTION

Living systems manifest many apparently deterministic behaviours at the macroscopic level, yet the molecular reactions upon which they are based are generally stochastic in nature. There has been increasing research on noise in the gene expression pathway, including regulatory steps, which can involve very small numbers of regulatory molecules in each cell. It is likely that the heterogeneity generated across cell populations by gene expression noise is utilized as a component of certain survival strategies (1). Indeed, stochasticity across the transcriptomes and proteomes of living organisms is likely to play important roles in cellular auto-regulatory circuits, phenotypic variation, cellular differentiation, stress responses, synchrony in circadian clocks, and probabilistic fate decisions such as viral latency (2–8). Noise also plays a role in evolution (9–12). On the other hand, noise is a potentially damaging source of imprecision, for example impacting on signaling and regulation (13–16), and evidence is emerging that living systems use multiple mechanisms to keep the level of randomness under control. Overall, it has become apparent that an appreciation of stochasticity in gene expression is essential to a full understanding of biology. However, there is still much work to do before we understand the full impact of noise as well as the overall picture of noise management in living systems.

It is now common to identify two overall classes of noise: intrinsic noise that is attributed to inherent stochasticity of expression from a specified gene system, and extrinsic noise that results from fluctuations in the intracellular environment, for example linked to the cell cycle and/or changes in the capacity of the expression machinery (17–19). Stochastic variations in the expression of reporter genes encoding fluorescent proteins are reflected in heterogeneity in the levels of these proteins in individual cells. A model of intrinsic noise predicted that prokaryotic cells would manifest higher levels of noise if transcription of a reporter gene was limited to low rates (20). Work in *Bacillus* found a positive correlation between translation efficiency and noise generation, so that a combination of weak transcription and efficient translation generates a relatively high level of noise (21). A comparable study in the yeast *Saccharomyces cerevisiae* found that noise strength for *GFP* gene expression increased linearly with translation efficiency (varied by changing codon usage) (22). Two studies in yeast have indicated that intrinsic noise scales inversely with protein abundance (23,24). In contrast, the level of observed intrinsic noise for mammalian cells does not always show this relation at lower protein abundance values (25). Other work has suggested that a high tRNA adaptation index (a measure of the relationship between gene codon usage and intracellular tRNA abundance in the context of different degrees of selection on translation efficiency) is correlated with noise (26).

Overall, most of the work on gene expression noise in eukaryotes (predominantly in the form of high-throughput genome-wide studies) has emphasized the influence of variations in mRNA copy number per cell that are driven by fluctuations in transcription, whereby correlations have been identified between noise level and gene characteristics such as promoter structure, gene function and chromatin density (16,24). The potential for posttranscriptional steps of gene expression, including translation, mRNA decay and protein degradation, to act as generators of noise, has received much less attention, and no investigation of possible mechanisms has been reported. However, gaining insight into the contributions of these steps is essential if we are to understand fully the landscape of noise generation across the genomes of living organisms. In the light of earlier work on the impact of inhibitory structures on translation initiation, we decided to examine whether noise-generating mechanisms can exist in this step of gene expression. This led us to consider how a combination of multiple sources of noise along a eukaryotic expression pathway impacts upon overall system behaviour. We have dissected out the contributions of transcription and translation by making measurements of both protein and mRNA abundance in single cells of *S.cerevisiae*, finding that translation-related stochasticity is an important contributor to overall noise. We discuss the mechanistic and wider biological implications of our data and also consider their significance for the field of synthetic biology.

## MATERIALS AND METHODS

### Strain construction

Strains used in this study were all derived from the background strain PTC830: *MATα ura3-1 leu2-3, 112 his3-11, 15 can1-100* (a derivative of W303). Genomic integration at the yeast *HIS3* locus was achieved via a plasmid containing the *KanMX* gene (encoding resistance to G418) and the reporter gene flanked by regions homologous to the 5΄ and 3΄ regions of the yeast *HIS3* open reading frame. Each of the modified plasmids was linearized (double-cut outside of the inserted sequence using PvuII or BglI, depending on the reporter), and then used for yeast transformation (leading to homologous recombination). A strains table is provided in the Supplementary Data section.

### Single molecule fluorescence *In situ* hybridization

smFISH was performed using custom Stellaris^®^ Quasar570-tagged probes directed against the yEGFP coding sequence and Quasar670-tagged probes directed against MS2 stem–loop repeats inserted into the 3΄UTR following a protocol adapted from previous work (27,28). The MS2 stem–loop repeats were originally intended for use in live-cell imaging, but we found this approach to lack consistency and accuracy in yeast (see full explanation in the Supplementary Data section) and decided to focus on smFISH instead. Two days prior to an experiment, single colonies from each of the strains were picked and grown overnight in YNB (plus amino acids, 2% glucose) to saturation with shaking at 30°C. The following morning, cells were diluted to give an optical density at 600 nm (OD_600_) of ∼0.1 and incubated further to an OD_600_ of 0.8–1.0. The cultures were then diluted again (via a serial dilution procedure) to the theoretical equivalent of OD_600_ = ∼0.0001 (i.e. ∼3 × 10^3^ cells ml^−1^) and allowed to grow overnight to an OD_600_ of 0.1–0.2 in 45 ml volume of minimal medium. Cells were then fixed by addition of 5 ml of 37% formaldehyde followed by incubation for 45 min. From this point onwards, all reagents and materials used were RNAse-free. Cells were washed twice with 1 ml ice-cold buffer B (1.2 M sorbitol, 0.1 M potassium phosphate, pH 7.5), centrifuged for 1 min to pellet the cells in between each wash. Cells were then converted to spheroplasts by resuspension in 1 ml of spheroplasting buffer (buffer B, 2 mM Vanadyl Ribonucleoside Complex, 250 U lyticase, 1:500-diluted 2-mercaptoethanol) and incubation for 25 min at 37°C. Cells were then washed twice with 1 ml ice-cold Buffer B, pelleted at low speed (for this and all the subsequent steps centrifugation was performed at 1300g/4°C for 5 min), resuspended in 1 ml 70% ethanol, and stored at –20°C until they were used for hybridization.

For each hybridization experiment, ∼200 μl of cells (adjusted according to the final OD_600_ value before fixation) were transferred into RNAse-free microcentrifuge tubes, centrifuged, and the ethanol was removed. Cells were incubated in 1 ml of wash buffer (10% formamide in 2× saline-sodium citrate (SSC) buffer) at room temperature in the dark for 2–5 min. Cells were pelleted again and resuspended in 100 μl of hybridization solution (100 mg/ml dextran sulfate, 10% formamide in 2× SSC buffer) containing a mixture of the two probe sets. The final probe concentrations were 100 nM for P*_TEF1_* constructs, 50 nM for P*_PAB1_* constructs and 25 nM for P*_DCD1_* constructs. Cells were incubated overnight in the dark at 30°C. On the following day, a chambered coverglass (Grace Biolabs, four wells) was incubated with 100 μl 0.01% poly-l-lysine/well at room temperature for 5 min. The solution was aspirated off, the coverglass was left to dry, each well to be used was washed 3× with nuclease-free water (100 μl) and allowed to dry again. Cells were washed with 1 ml of wash buffer (10% formamide in 2× SSC buffer), resuspended in another 1 ml of wash buffer and incubated at 30°C for 30 min in the dark. To stain the cell nuclei, cells were resuspended in 100 μl of 0.01 μg/ml 4΄,6-diamidino-2-phenylindole (DAPI, as a solution in 2× SSC), loaded on poly-l-lysine-treated chambered coverglasses and incubated for 30 min in the dark at 30°C. Cells were briefly washed in 100 μl 2× SSC/well, incubated in 100 μl GLOX buffer (0.4% glucose in 10 mM Tris, 2× SSC) for 1–2 min at room temperature in the dark. GLOX buffer was removed and 80 μl of GLOX buffer containing glucose oxidase and catalase was added to each sample. A clean slide was placed over the wells to spread the GLOX buffer over the entire sample and prevent evaporation. The imaging acquisition and analysis procedures are described in the Supplementary Data section.

### Flow cytometry

Cells were prepared for flow cytometry as described in the Supplementary Data section. Yeast cells expressing the yEGFP or ymNeonGreen reporter genes were excited using a 488 nm laser, and fluorescence was collected through 505 nm long-pass and 530/30 nm band-pass filters on a BD Fortessa X20 flow cytometer. For dual-colour reporter strains, yEGFP was excited and fluorescence was collected using the same laser and filters as described above while mRuby3 was excited using a 561 nm laser and its fluorescence collected through a 600 long-pass plus 610/20 nm band-pass filters. The data were recorded using the ‘Area’ option. Flow cytometry data were exported from the acquisition program (FACSDiva) in the FCS3.0 format with a data resolution of 2^18^. A custom R programme was written (using flowCore, flowViz and flowDensity Bioconductor packages; see Supplementary Data section) to calculate statistics for each file. For calculating the coefficients of variation, cytometry files were processed as follows:

1. The first second, and final 0.2 seconds, of data were removed to minimize errors due to unstable sample flow through the cytometer.
2. Thresholds of 40 000–100 000 and 10 000–90 000 for the FSC and SSC gates, respectively, were typically used to limit the influence of cellular debris and aggregated cells.
3. For the remaining data, the FSC and SSC values of the highest density centre of the FSC–SSC scatterplot were calculated, and the distance of the *i*th sample to the centre was determined:
  \begin{equation*}\begin{array}{@{}*{1}{l}@{}} {{\rm{Distance}}\;{{i}}}\\ { = \surd ({{({\rm{FSC}}\;{{i}} - {\rm{FSC}}\;{\rm{centre}})}^2} + {{({\rm{SSC}}\;{{i}} - {\rm{SSC}}\;{\rm{centre}})}^2})} \end{array}\end{equation*}
4. The fluorescence reporter data within the radius were used to calculate the coefficient of variation, i.e.
  \begin{equation*}{\rm{CV}} = {\rm{s}}/{\rm{m}}.\end{equation*}

yEGFP (ymNeonGreen) data were obtained from 10 (six) independent experiments, whereby the centre point for the scatter plot analysis was either set automatically, or manually at FSC = 59 000/SSC = 27 000. The average number of cells analyzed given a radius limit of 4000 was ∼780 (900). This gate radius was chosen as a compromise point at which, over multiple experiments, the variation between experiments was minimal and the number of cells analysed provided statistically meaningful results. This procedure is similar to one reported previously (24) except that, by focusing on the cell density centre, we have been able to maximize the number of cells that are sampled.

In the two-reporter measurements (6 independent measurements), the centre was set at FSC = 57 000/SSC = 24 500, and the average number of cells contained in the final gate was 961. In order to calculate the intrinsic, extrinsic, and total noise from dual-color flow cytometry data we sought to identify an appropriate normalization procedure. Comparative assessment of two approaches to this challenge (Supplementary Figure S4) led us to follow the statistical analysis procedure described elsewhere (24) in the evaluation of our data. The R script used to enable automatic processing of the data is given in the Supplementary Data section. In the Supplementary Data section, we also discuss the influence of reporter gene structure on absolute noise value estimates.

#### Computational modeling

Details of the model and its outputs are given in Figure 7, the SI Appendix, and in Supplementary Figure S6. Simulations were carried out using the Gillespie stochastic simulation algorithm (29) implemented in the software COPASI (30).

## RESULTS

### Genomic expression constructs designed to modulate translation and transcription

**Figure 1.**
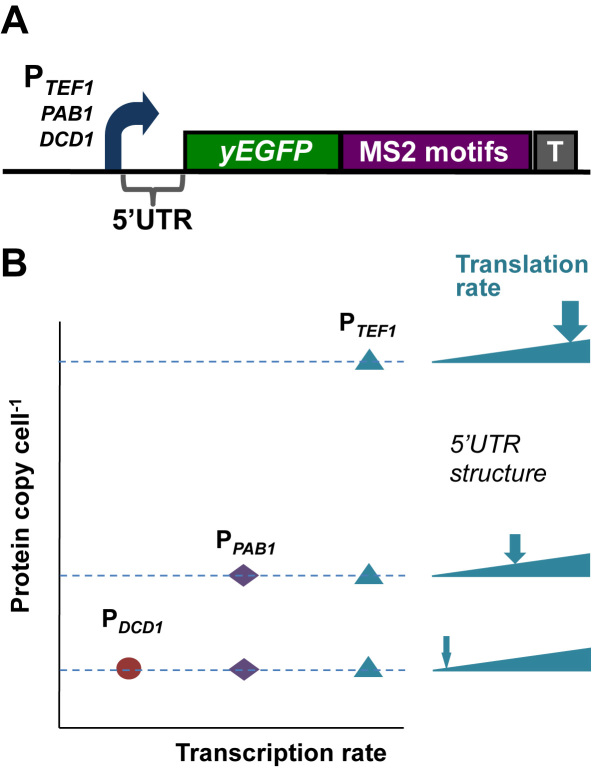
Chromosome-integrated reporter constructs. (**A**) The integrated reporter (in this example, yEGFP) constructs were transcribed by yeast promoters of differing strengths: P*_TEF1_*, P*_PAB1_* or P*_DCD1_*. A range of 5΄UTRs was used to dictate different translation initiation rates. 24 bacteriophage MS2 binding motifs were added to the 3΄UTR as additional targets for smFISH probes. The *PGK1* terminator (T) was introduced at the end of the string of MS2 motifs. (**B**) Concept of combining different-strength promoters with a range of 5΄UTRs to create a spectrum of ratios of transcription vs translation. For example, the overall expression rate driven by P*_TEF1_* combined with a non-structured 5΄UTR (L0) can be reduced to the rates of overall expression supported by P*_PAB1_*-L0 or P*_DCD1_*-L0 by inserting structured 5΄UTRs.

In order to analyze the respective contributions of transcription and translation to gene expression noise in *S.cerevisiae*, we built genomic constructs whose expression rate is subject to restriction at two different points in the expression pathway (Figure 1A). We chose to use a small number of reporter genes (rather than a large number of reporter fusions with endogenous genes) in order to avoid having to measure (and correct for) variations in the stabilities of the respective gene fusion mRNAs and proteins. For consistency, all three of the promoters we used lack TATA boxes, since these elements have been reported to contribute to increased noise levels by affecting transcriptional burst size (31,32). Starting at the upper range of transcription, a strong constitutive promoter (P*_TEF1_*) generates a comparatively large number of mRNA molecules per cell. Guided by previous work (33–35), we then attenuated the overall expression rate for each of these mRNA molecules by inserting into the 5΄UTR structural elements that limit translation initiation to different degrees. Three types of structural element were introduced: stem–loops of different stabilities, two different lengths of poly(G), and an upstream open reading frame (uORF). We also built constructs in which transcription was driven by a mid-range promoter (P*_PAB1_*) or a weak promoter (P*_DCD1_*) (Figure 1B). In this way, we planned to achieve a low rate of expression both via a high-transcription/low-translation combination and via a low-transcription/high-translation combination. We assessed the inhibitory impact of the 5΄UTR structures (Supplementary Data section and Supplementary Figure S1) in order to identify the range of inhibitory structures that provide the required spectrum of transcription/translation ratios (Figure 1B; Supplementary Table S1).

### Transcriptional noise for three promoters of different strengths

**Figure 2.**
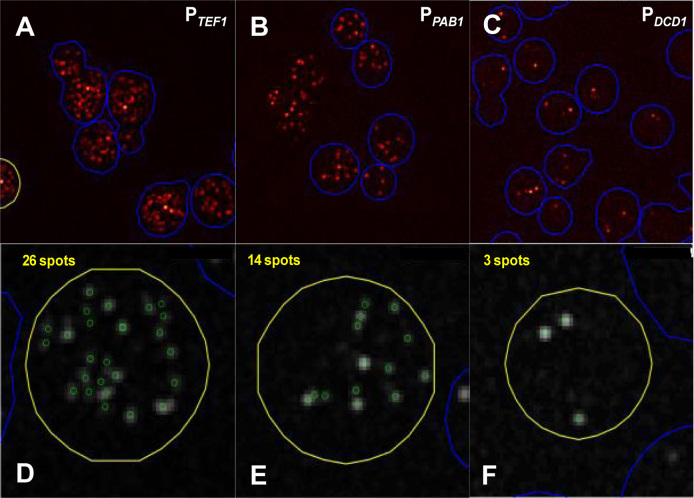
smFISH measurements of mRNA copies per cell. (**A–C**) Randomly selected images of cells revealing foci labeled with Quasar 670—tagged probes targeted to the multiple bacteriophage MS2 motifs in the 3΄UTRs. (**D–F**) mRNA foci in individual cells identified and counted as described in the Supplementary Data section.

**Figure 3.**
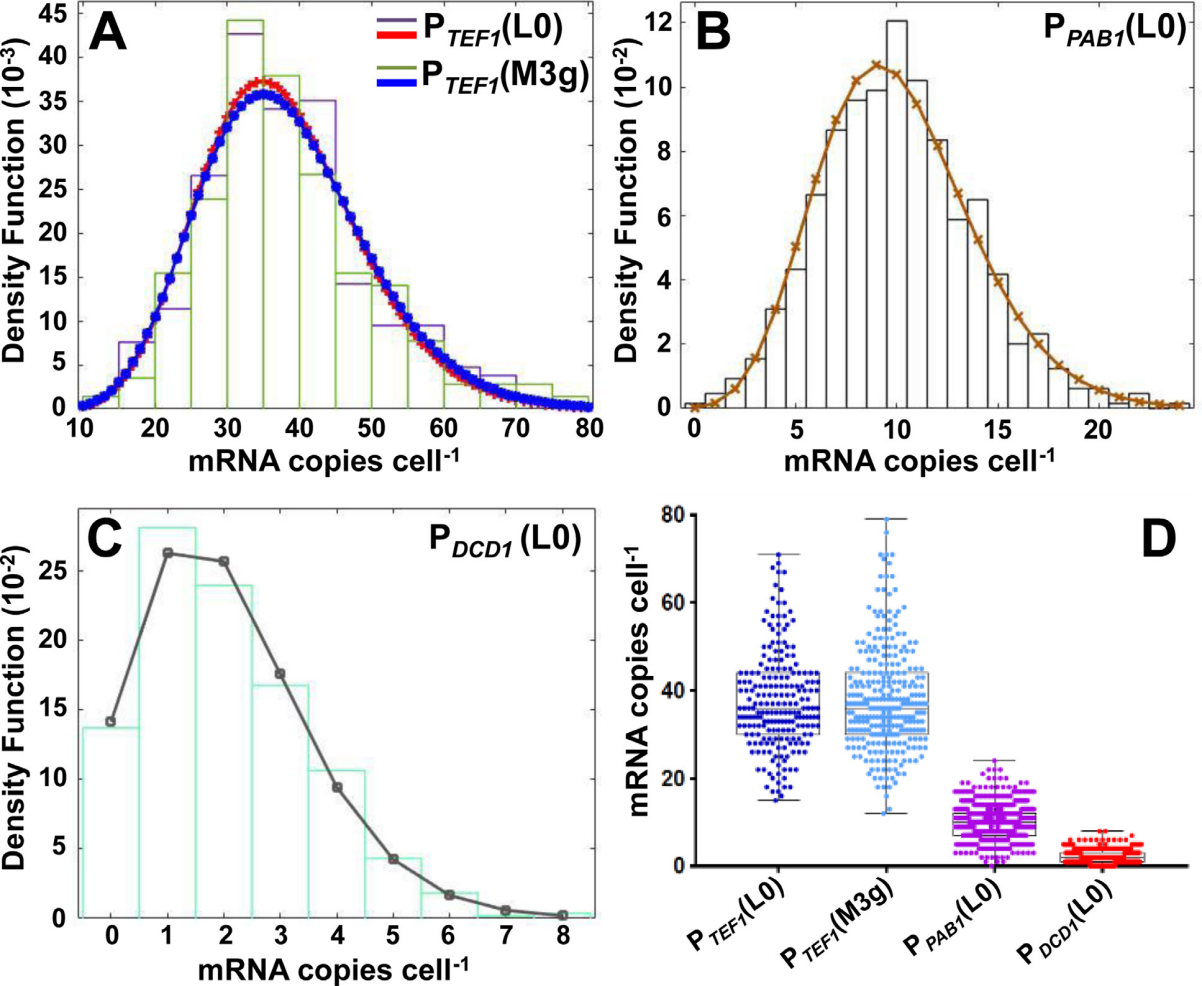
Distributions of mRNA (yEGFP) counts per cell. (**A**) Overlaying the distributions for P*_TEF1_*(L0) and P*_TEF1_*(M3) reveals no significant effect of inserting the M3 stem–loop structure into the 5΄UTR. Panels **B** and **C** show the corresponding data for P*_PAB1_*(L0) and P*_DCD1_*(L0), respectively. (**D**) Overview of the data shown in A–C. The key statistical data are summarized in Table 1. The data obtained with the P*_TEF1_* and P*_PAB1_* promoters fit to negative binomial distributions (A, B). In the case of P*_DCD1_*(**C**), it is difficult to distinguish between negative binomial and Poissonian fits.

We compared the basic properties of the three promoters. Single molecule fluorescence *in situ* hybridization (smFISH) was used to monitor the level of reporter mRNA generated in each cell of the strains created in this study. In order to enhance the intensity of the FISH signals, and thus the sensitivity (as well as accuracy and precision) of detection of intracellular RNA molecules, we incorporated 24 copies of the bacteriophage MS2 coat protein binding motif into the 3΄UTR of the genomic reporter construct since this allowed us to achieve a higher signal intensity with the smFISH probes (Figures 1 and 2 and Supplementary Data section). Examination of the smFISH data for the three promoters combined with the (unstructured) control 5΄UTR revealed mean mRNA copies per cell of 38 (P*_TEF1_*), 10 (P*_PAB1_*) and 2.0 (P*_DCD1_*), respectively (Table 1 and Figures 2 and 3). The mRNA copy numbers per cell across each cell population fit either unequivocally to a negative binomial distribution (P*_TEF1_* and P*_PAB1_*) or fit to a distribution that appears to lie somewhere between negative binomial and Poisson (P*_DCD1_*). However, in the latter case the exact nature of the distribution is less easily judged because the mean is so close to zero (Figure 3). These results are consistent with stochastic fluctuation between promoter on and off states (36), but do not exclude the operation of other models in which the promoter may manifest multiple levels of activity (37,38). As expected, the coefficient of variation (CV) for mRNA copy number per cell decreases with increasing promoter strength (compare, for example, the L0 constructs in Table 1).

**Table 1.** mRNA and protein noise values

Construct	mRNA copies per cell			Reporter fluorescence per cell*		Reporter fluorescence per cell*	
yEGFP						ymNeonGreen^#^	
	No. cells (smFISH)	μ	CV (%)	μ	CV (%)	μ	CV (%)
**P*_TEF1_***							
L0	211	38 ± 11	29.5	1560	11.8 ± 0.5	3050	12.2 ± 0.3
U	–	nd	nd	526	12.4 ± 0.6	nd	nd
M1Ug	526	35 ± 10	28.3	408	13.1 ± 0.6	nd	nd
M3g	285	38 ± 12	30.8	226	16.0 ± 0.4	nd	nd
M3Ug	394	37 ± 9.5	25.8	ea	ea	nd	nd
G_10_	417	37 ± 10	27.1	448	12.9 ± 0.5	nd	nd
G_14_	313	63 ± 16	25.9	ea	ea	317	15.4 ± 1.5
M3Wn	–	nd	nd	nd	nd	1143	13.1 ± 0.8
M3n	–	nd	nd	nd	nd	579	13.3 ± 0.5
M3Un	–	nd	nd	nd	nd	377	13.9 ± 0.6
**P*_PAB1_***							
L0	647	10 ± 3.8	37.8	288	13.8 ± 0.2	495	13.7 ± 0.4
M1Ug	324	10 ± 4.2	41.6	ea	ea	nd	nd
G_10_	316	10 ± 4.1	41.2	ea	ea	nd	nd
**P*_DCD1_***							
L0	554	2 ± 1.5	73.4	ea	ea	150	21.3 ± 0.8^†^

*These are gated values for protein fluorescence (including a defined number of cells; see Supplementary Data section).

^#^The stem–loop structures (M3Wn, M3n and M3Un) are similar, but not identical, to those inserted

upstream of yEGFP (suffixed with g in Supplementary Figure S1).

^†^Partial overlap with endogenous autofluorescence.

ea: not calculated because of overlap with autofluorescence.

nd: not determined.

### Modulation of mRNA cell-to-cell heterogeneity by structure in the 5΄UTR

The three types of structural element mentioned above were introduced into the 5΄UTR in order to impose different combinations of transcription rate and translation rate (Figure 1 and Supplementary Figure S1). Minor sequence adjustments were introduced to maintain a comparable inhibitory capacity for each stem–loop structure as we switched from one reporter gene to another (Supplementary Figure S1). Each upstream AUG was engineered to create an uORF that overlaps with, and terminates within, the reporter gene ORF in the +1 reading frame. Overlapping uORFs are known to occur in a number of natural eukaryotic transcripts (39 and references therein). In addition, in two constructs a stem–loop (either M1 or M3) was combined with the same uORF (creating M1U and M3U), thus reflecting combinations of multiple translation-inhibiting elements that are known to occur in natural mRNAs.

We assessed whether the cell-to-cell variation in intracellular mRNA abundance was affected by our set of 5΄UTR structural elements. There was a striking consistency in both the mean abundance and the variation in abundance across the respective P*_TEF1_* and P*_PAB1_* constructs (Table 1). Figure 3A strikingly illustrates the absence of any significant effect of stem–loop structures on mean mRNA abundance or copy-number heterogeneity. On the other hand, it has been demonstrated previously that a poly(G) sequence (G_18_) blocks the 5΄-3΄ exonuclease activity of Xrn1, thus leading to the accumulation of deadenylated and decapped mRNAs (40,41; Supplementary Figure S2). Recent work has shown that a continuous sequence of guanines in DNA manifests proton NMR spectra indicative of higher order structure once the number of Gs reaches 12 or more (forming G-quadruplexes, four-stranded helical structures held together by a guanine core; 42). In this study, we have used one poly(G) sequence that is shorter (G_10_) than this threshold length, and one that is longer (G_14_). The smFISH data indicate that the G_10_ motif has little effect on mRNA abundance, whereas G_14_ has a major impact. It is notable that the CV value for transcript abundance is not significantly affected in response to incorporation of G_14_ (Table 1), despite the fact that the copy number is increased (overall by approximately 1.8-fold) by virtue of a reduced rate of 5΄-3΄ exonucleolytic mRNA degradation.

### 5΄UTR structure promotes increased noise

**Figure 4.**
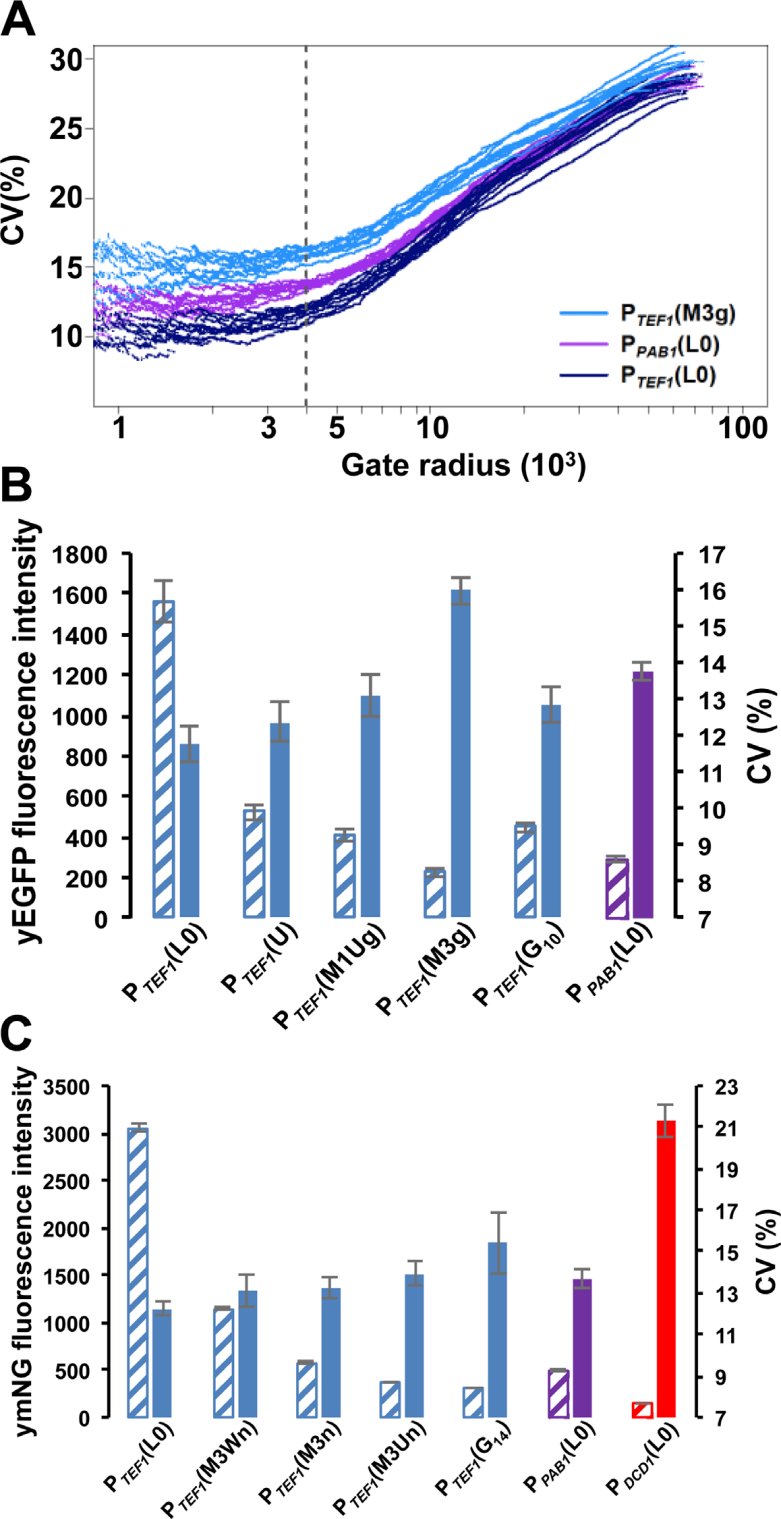
Gated flow cytometry data for reporter constructs. (**A**) Analysis of flow cytometry data to determine the relationship between the gate radius and CV(%) for constructs containing yEGFP. Ten experiments were performed for each of the genomic constructs; this panel illustrates the variation in CV values for three of these constructs. Using a gate radius of 4000 (dashed vertical line in panel A), we obtained plots of CV(%; solid bars) versus mean fluorescence intensity values (hatched bars) for yEGFP (**B**) and for ymNeonGreen (**C**) constructs. The greater fluorescence intensity of ymNeonGreen enabled us to distinguish fully the fluorescence intensity distributions of P*_TEF1_*(M3Un) and P*_TEF1_*(G_14_) from host cell autofluorescence, and to distinguish partially the distribution for P*_DCD1_*(L0) (Supplementary Figure S3).

Previous results have suggested that under conditions of active translation, mRNAs with structured 5΄UTRs might interconvert dynamically between sub-populations with folded and unfolded stem–loops, respectively (43, 44). We accordingly tested the hypothesis that the structural elements inserted into the 5΄UTR of our genomic constructs (Figure 1 and Supplementary Figure S1) could act as noise generators at the level of translation. Our assessment of the degrees of inhibition imposed by different structural elements (Supplementary Figure S1) allowed us to identify which of the structures tested would constrain the overall expression rate from the P*_TEF1_* promoter to match the rates of the P*_PAB1_* and P*_DCD1_* promoters. We selected from the promoter/5΄UTR combinations tested as described in Supplementary Figure S1 a subset that were then placed upstream of the yE*GFP* reporter gene (Table 1 and Supplementary Table S1). However, as a result of wanting to examine the widest possible range of reporter expression rates, we found that the yEGFP fluorescence intensity profiles of some of our weakest expressing constructs were not fully resolved from the endogenous autofluorescence emission profiles of the host cell (Supplementary Figure S3). We therefore performed parallel experiments using a yeast-optimised version of the recently described intensely fluorescing reporter mNeonGreen (45; Table 1) in order to eliminate uncertainty about the expression characteristics of the weaker constructs (Figure 4; Supplementary Figure S3). We also performed other technical controls to verify the reliability of the flow cytometry measurements (Supplementary Figure S3).

Cell-to-cell heterogeneity in a non-synchronized population of cells will capture a range of distinct cell states. Part of this overall picture is that variations in the activities of components of the machineries that are responsible for gene expression will contribute to variations in the rate at which cells progress through the cell cycle (46). In order to understand how these extrinsic factors contribute to the cell-to-cell heterogeneity observed in our experiments, we utilized a modified version of the approach described previously in which noise in flow cytometry data is assessed as a function of the gating radius centred around the medians of the forward (FSC) and side (SSC) scatter parameters (24; Supplementary Data section). This procedure takes advantage of the fact that scattering parameters reflect the physical heterogeneity of cell populations, allowing selection of cell subpopulations that are less varied in terms of cell shape, size and cell-cycle stage, thus reducing the contribution of extrinsic factors to overall noise. The outcome of such analysis in this context is that it highlights principally the intrinsic component of the total noise.

Seen in the context of the minimal differences in transcript abundance heterogeneity across the respective constructs, examination of these flow cytometry data reveals that the introduction of stable secondary structures into the 5΄UTR causes posttranscriptional gene expression noise (Figure 4, Table 1). For example, comparison of P*_TEF1_*M3 with the other genomic constructs reveals that the M3 stem–loop structure, whether alone or combined with an uORF (as in M3U), causes increased noise relative to the control mRNA lacking added secondary structure (P*_TEF1_*L0) and also relative to constructs in which the 5΄UTR contains a less stable secondary structure (P*_TEF1_*M1, P*_TEF1_*G10). It is also notable that the G_14_ element (P*_TEF1_*G_14_), which strongly inhibits both the Xrn1 exonuclease and the scanning ribosome, causes a major increase in noise (as measured with ymNeonGreen). Moreover, insertion of either M3 or G_14_ into the 5΄UTR downstream of the P*_TEF1_*promoter can generate gated noise values that are equal to or greater than those measured for P*_PAB1_*L0 (Table 1).

Very low transcription of the mNeonGreen gene from the P*_DCD1_*L0 construct results in a reporter fluorescence profile that overlaps with host autofluorescence (Supplementary Figure S3). Accordingly, the result obtained with P*_DCD1_*L0 allows us to make a less precise gated estimate of cell-to-cell heterogeneity for the encoded mNeonGreen reporter protein of ≤0.21 (21%). Moreover, the trend in CV values observed as we increase the inhibitory impact of structures inserted into the 5΄UTR indicates that, if we could measure accurately the noise associated with inhibitory structures even more stable than M3 and G_14_, these would extend into the range 0.15-0.20 (15-20%). Overall, while noting that the absolute total (protein) noise values for yEGFP and ymNeonGreen will be influenced by the degradation rates for these respective reporter proteins (see Discussion of the influence of reporter structure in the Supplementary Data section), we can see a consistent enhancement of noise by translational inhibition (Table 1). Indeed, these data reveal that, in the presence of inhibitory structures in the 5΄UTR, additional noise is generated that is of similar magnitude to the noise enhancement observed when switching from a strong promoter (P*_TEF1_*L0) to a much weaker promoter (P*_PAB1_*L0, P*_DCD1_*L0).

### Differentiation of intrinsic and extrinsic noise components

**Figure 5.**
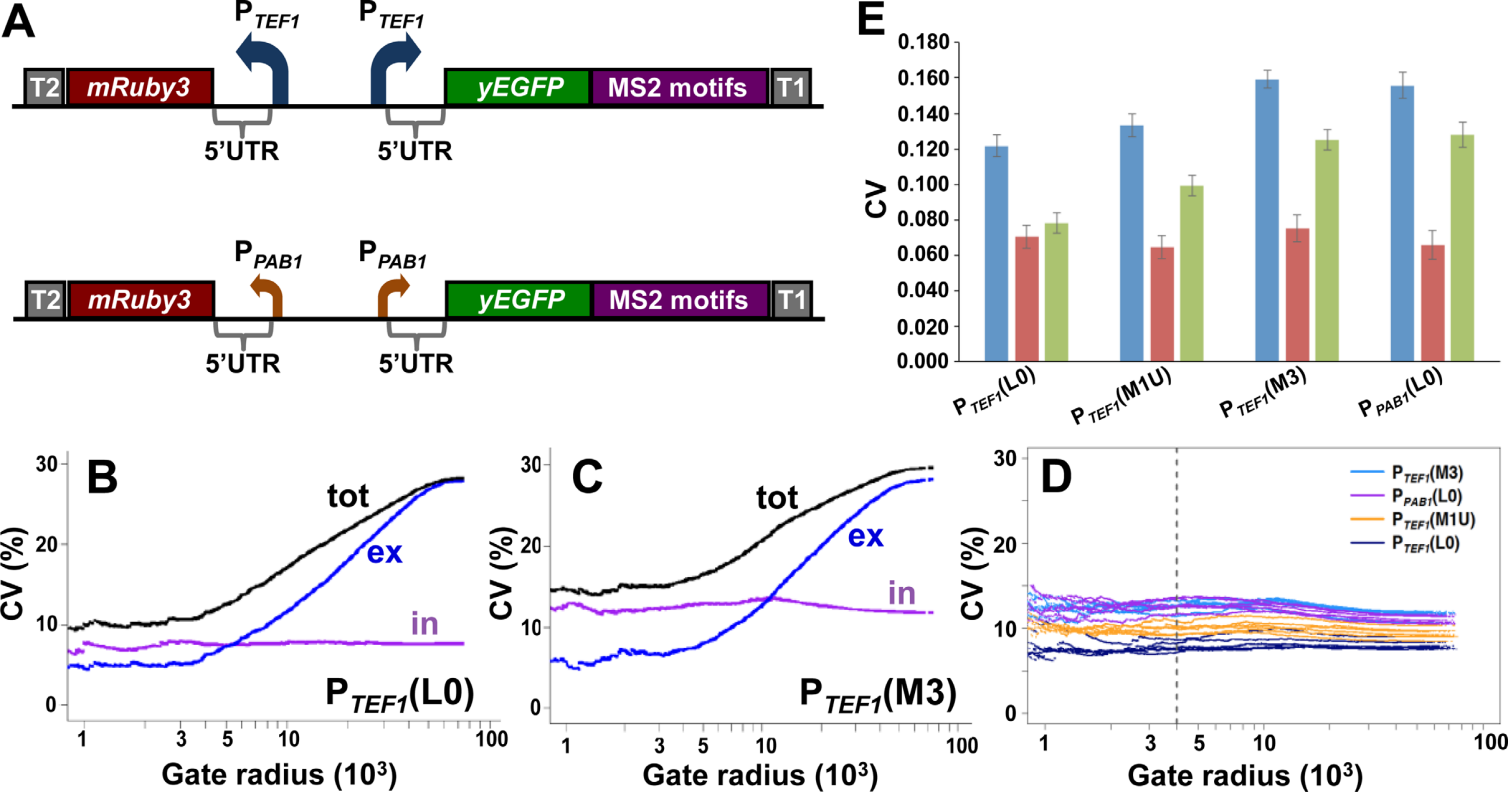
Dual reporter analysis of gene expression noise. Methodological details are described in the Supplementary Data section. (**A**) Overall design of the genomic dual reporter constructs. Each *yEGFP* construct (see Figure 1) was combined with a second, oppositely oriented construct that was identical except that the *yEGFP* gene was replaced by the *mRuby3* gene. Plots of intrinsic, extrinsic and total noise versus gate radius show exemplar data from single experiments with the dual (P*_TEFI_*-transcribed) reporter constructs bearing the 5΄UTRs L0 (**B**) and M3g/r (**C**). Panel **D** shows the relationship between gate radius and intrinsic noise from six repeat experiments performed with each of the dual reporter constructs (colour-coded). The vertical broken line corresponds to a gate radius of 4000, which defines the subset of cells whose fluorescence data are used for comparative noise analysis. (**E**) Summary of the results obtained from all of the experiments (data shown in Supplementary Table S2), showing average values and standard deviations for total noise (blue bars), extrinsic noise (red bars) and intrinsic noise (green bars). In each construct, the same (indicated) 5΄UTR is inserted upstream of both of the reporter genes.

**Figure 6.**
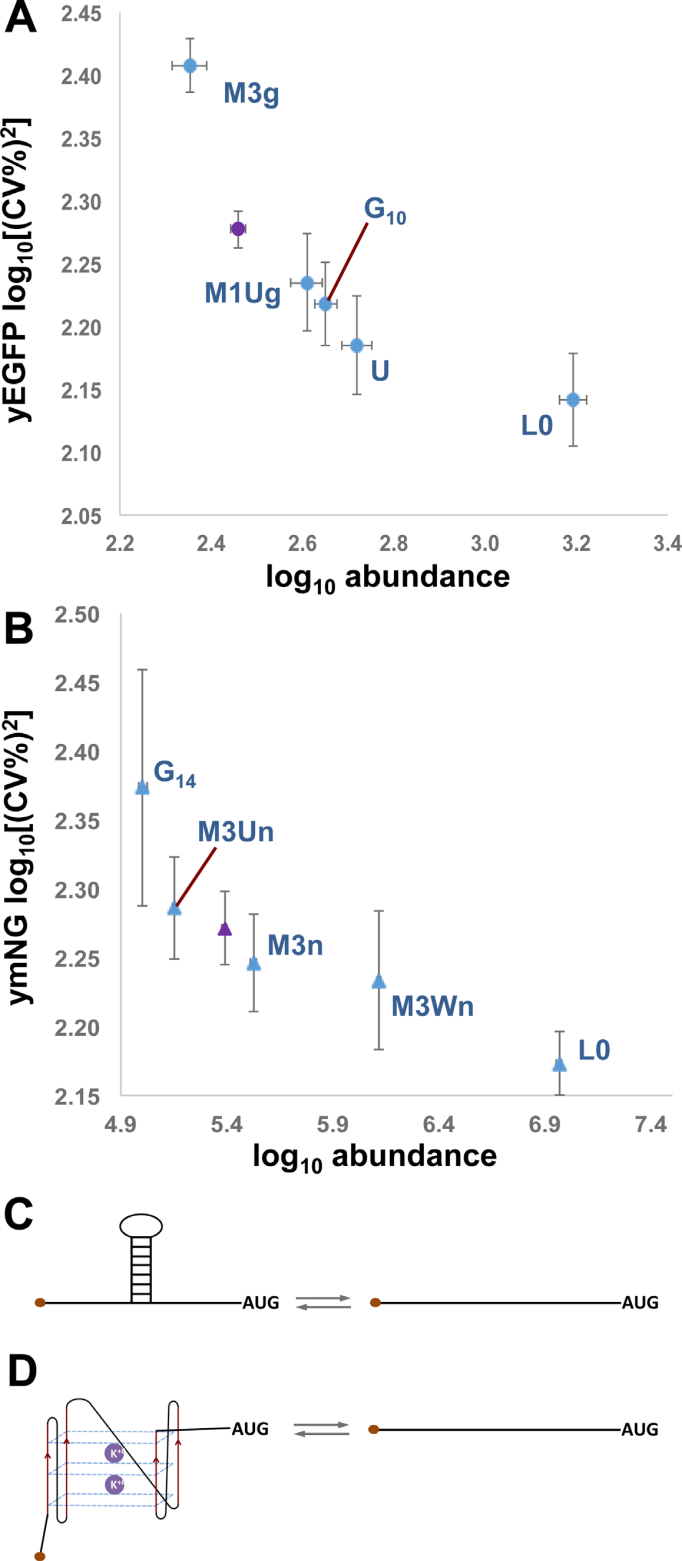
Gene expression noise in relation to 5΄UTR structure. Log_10_ mean fluorescence vs log_10_ gated noise strength (CV^2^) for yEGFP (**A**) and ymNeonGreen (**B**) genomic constructs. Standard deviation values are represented by the error bars on the plots. The data points in blue correspond to P*_TEF1_* constructs, and are labeled according to the structural elements inserted into the 5΄UTR. The purple data point in each panel corresponds to P*_PAB1_*(L0). Schematic drawings illustrate potential stochastic conformational interconversions of a stem–loop structure (**C**) and of a poly(G) structure (**D**).

We wanted to obtain more accurate information about the intrinsic and extrinsic components of gene expression noise observed with the respective reporter mRNAs. Following earlier work (13), this involved the characterization of the expression ratio between two constructs that have identical promoters and 5΄UTRs but different reporter genes (*yEGFP* and *mRuby3*). Since extrinsic noise factors affect the two constructs simultaneously and in principle equally, the ratio between their expression levels reflects the intrinsic noise components. We chose to build back-to-back genomic expression constructs in order to perform this analysis on a range of our 5΄UTRs (Figure 5A). In each construct, two independently acting copies of a promoter (either P*_TEF1_* or P*_PAB1_*) were arranged in a divergent orientation in order to avoid any transcriptional interference (which can only occur when two promoters are configured to be convergent, tandem or overlapping; 47). There is a striking consistency in mean fluorescence intensity and CV values for yEGFP in single- and dual-reporter configurations (Figures 4 and 5; Table 1 and Supplementary Table S2; Supplementary Data section). However, the most remarkable feature of the dual reporter data is that they highlight the impact of 5΄UTR structure on the intrinsic component of gene expression noise. In particular, a stable stem–loop structure (e.g. M3) is seen to boost the intrinsic noise component. The gated data follow the same trend in terms of noise (Figure 4; Supplementary Table S2), and there is a marked inverse proportionality between the gated measurements of mean fluorescence and CV^2^ (as well as CV) for the respective genomic constructs (Figure 6 and Supplementary Figure S5). This is consistent with the results of an earlier proteome-wide analysis in *S.cerevisiae* (24), although in the case of our data the different noise levels are specifically linked to translation events. Our analytical procedure (Supplementary Data; Figure S4) incorporates independent normalization of the data sets for the respective reporter genes.

### Modelling ribosomal scanning noise

**Figure 7.**
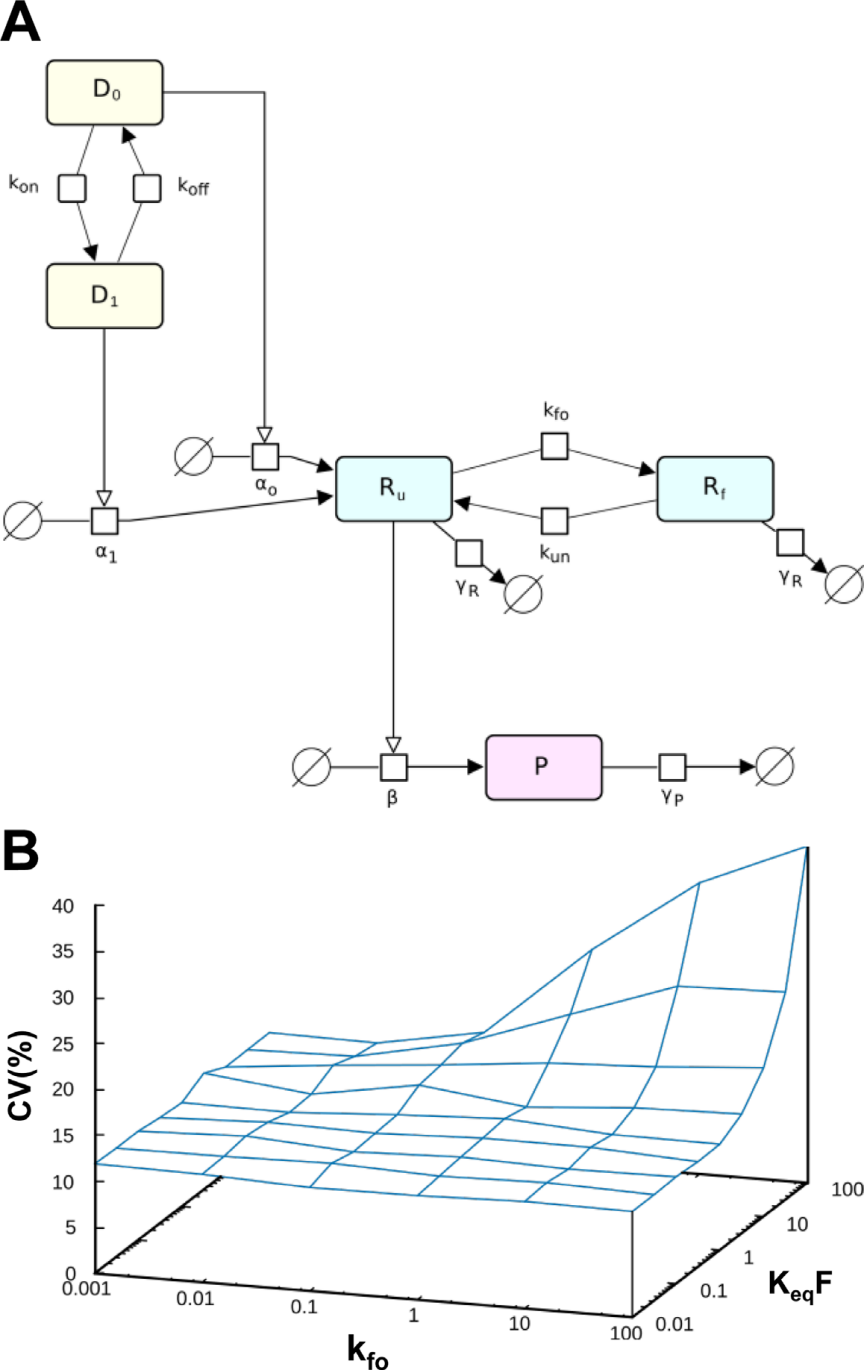
Computational modeling illustrates how translational bursting could generate noise. (**A**) A scheme showing reversible stochastic events in the gene expression pathway, featuring promoter on (D_1_) and off (D_0_) states) and mRNA (5΄UTR) folded (R_f_) and unfolded (R_u_) states. P is protein, and γ_R_ and γ_P_ are degradation rates for mRNA and protein, respectively. (**B**) Predicted dependence of noise in protein level as a function of the kinetic parameters of mRNA folding/unfolding.

We investigated the ability of a suitably formulated model incorporating mRNA folding/unfolding intended to represent inhibition of ribosomal scanning events. Starting from a previously reported model (15), we have incorporated a folding step that allows reversible formation of an inhibitory secondary structure in the mRNA 5΄UTR (Supplementary Data section, Figure 7 and compare Supplementary Figure S6). Inclusion of this step allows us to simulate the contribution of stochastic translation inhibitory events to overall gene expression noise. We have explored how selection of the parameters for folding/unfolding of secondary structure affects the predicted behaviour of the system. Both the predicted thermodynamic stability of a stem–loop, and the folding/unfolding kinetics, are predicted to influence noise generation. Computational modelling thus illustrates how expression stochasticity driven by mRNA folding/unfolding can be as significant as promoter-driven noise (Figure 7; Supplementary Figure S6). Overall, the model provides a useful tool for predicting the impact of inhibitory elements on gene expression noise.

### Analysis of genome-wide expression and noise data

Our observations with reporter gene constructs lead to the testable hypothesis that a range of endogenous mRNAs with structured 5΄UTRs are likely to manifest translation-generated noise. We decided to analyse previously published data sets in order to extract information relevant to this question. This cannot be achieved simply by assessing expression data generated by published genome-wide measurements using reporter fusions, since this type of earlier work did not determine translation rates. We have therefore taken advantage of an alternative approach, based on the observation that the DEAD-box RNA helicase Ded1 is required for optimal translation of mRNAs bearing longer, more complex, 5΄UTRs (48). Translation efficiency measurements from a recent high-throughput study that highlights the impact of a *DED1* mutation on yeast mRNAs (49) identifies a group of mRNAs whose translation is restricted by their structured 5΄UTRs. We have compared these translation data with intrinsic noise estimates for equivalent genes obtained via a single fluorescent reporter colour approach that compares expression from one type of reporter (YFP) fusion present in either one or two copies in otherwise isogenic diploid yeast cells (50). It should be pointed out that there is a degree of uncertainty about the precise comparability of expression data in studies that have not determined the stabilities of the respective reporter-fusion mRNAs and proteins. Despite this uncertainty, we regard the outcome of this initial comparison as a useful indicator of whether there exists a trend in terms of a detectable relationship between 5΄UTR structure and noise. We find that a subset of mRNAs whose translation efficiency is strongly dependent on Ded1 manifests significantly higher mean noise values than those of the total group (see Supplementary Data section). Overall, increased dependence of translation efficiency on Ded1 activity correlates with increased intrinsic noise values. The data suggest that the magnitude of this effect is at least comparable with the impact on (transcriptional) noise of the TATA box (Supplementary Data section and refs 31,32).

## DISCUSSION

One route via which the translation process can contribute to gene expression noise is by amplifying the fluctuations in mRNA template abundance generated by varying promoter function: the translation machinery is thought to generate peaks of protein molecule abundance from the bursts of mRNA produced from each promoter. The amplification effect can be significant: for example, in exponentially growing yeast, a molecule of one of the more stable mRNA species (*t*_1/2_ ≥ 30 min) can act as the template for the production of >2000 protein molecules. The variations in protein abundance are expected to reflect the fluctuations in mRNA abundance in a relationship influenced by the rates of synthesis and degradation of protein as well as by the rate of cell growth (51). Indeed, it has been concluded elsewhere that observed correlations between codon usage and expression noise are related to the ability of translation efficiency to amplify transcriptional noise (26,52). Perhaps somewhat confusingly in this context, translation efficiency (the average number of protein molecules produced from each mRNA molecule) is commonly referred to as ‘translational bursting’ (20), which can be formalized as the product of a rate parameter and a duration parameter (related to decay and dilution rates; 52). In this scenario, it has been assumed that expression pulse duration is determined predominantly by transcription while translation is the dominant process in setting the amplitude.

Studies of the noise profiles of proteome-wide GFP fusions have previously revealed an inverse relationship between protein abundance and noise (23–25). However, these studies have generally not resolved the sources of the noise for the respective genes. Here, we have recreated this type of inverse relationship for individual genes by varying the degree of translational inhibition imposed by structural elements in the 5΄UTR (Figure 6 and Supplementary Figure S5). This suggests that the overall noise profiles of many eukaryotic genes represent the sum-total of contributions from both transcriptional and posttranscriptional mechanisms. Indeed, we find that the introduction of an inhibitory structure into the 5΄UTR can have a comparable effect on gene expression noise to that observed upon changing from a strong promoter to a weak-to-medium promoter. In other words, it is predicted that noise induced by translational inhibition can represent a significant component for those genes whose 5΄UTRs bear sufficiently stable structural elements. The results of our analysis of previously published data on structured mRNAs are consistent with this expectation, but the underpinning hypothesis requires dedicated experimental testing (see below). The existence of this type of mechanism for generating translational noise could help explain discrepancies that have been observed between predictions of noise behaviour from transcriptional bursting models and actual noise measurements (52).

Inhibitory structures are present in the 5΄UTRs of a sizeable subset of eukaryotic mRNAs that includes many regulatory mRNA species (53,54). Intriguingly, many of the hundreds of yeast mRNAs with highly structured 5΄UTRs have as yet uncharacterized functions (and could have regulatory roles; 55). Moreover, studies of the effects of synthetic stem–loop structures on the expression of reporter mRNAs have revealed a predictable relationship between the free energy of stem–loop folding/unfolding and the degree of inhibition imposed on translation initiation (54). Thus posttranscriptional noise generation of this type is likely to have broad significance in the context of the evolution of global gene expression profiles (56). Furthermore, uORFs can also affect the posttranscriptional control of gene expression in different ways, depending on their structure, length and position relative to the main ORF (54, 57). Recognition of the start codon of the type of uORF used in this study (Supplementary Figure S1) causes more than half of the scanning ribosomes to bypass the main ORF start in the +1 reading frame, thus reducing translation of the reporter gene by the equivalent amount. The fact that, in the absence of a stem–loop structure, such an uORF does not enhance noise suggests that stochasticity does not simply respond to changes in gene expression rate *per se*.

It seems likely that the mechanism underpinning translational noise generation involves repeated folding-unfolding cycles of each structural element inserted into the 5΄UTR (Figures 6 and 7). Interconversion between more or less stable higher-order structures will allow randomly timed bursts of scanning through this type of structural element. RNA helicases that are known to promote ribosomal scanning are likely to be involved in the structural rearrangements of both types of element. However, it is important to emphasise that we do not know the kinetics of interconversion of folded and unfolded states *in vivo*. We can imagine, for example, that the 5΄UTRs of a large proportion of a population of a certain species of mRNA might be blocked by a folded structural element for most of the lifetimes of these molecules. As a consequence, a 5΄UTR structural element may constrain the number of translationally active members of even a comparatively large mRNA population to a small number.

Poly(G) stretches occur quite widely in diverse genomes in a range of locations (for example in promoters, telomeres and 5΄UTRs; 42,58). The G_14_ motif selected for use in this study is likely to have an intermediate propensity to form a stable G-quadruplex structure (42), consistent with its ability to inhibit translation initiation in yeast by approximately 90% (Table 1; Supplementary Figure S1). This contrasts with the almost 100% inhibition observed with a G_18_ motif (34,41). The inhibitory influence of the G_10_ motif is detectable (Figure 4; Table 1), but its comparatively weak impact on translation (and lack of influence on mRNA stability) is consistent with its inability to form a stable G-quadruplex. Since the G_14_ element will not change the rates of deadenylation and decapping, the steady-state numbers of reporter mRNA molecules that are capped/adenylated and capped/deadenylated are unlikely to be changed by the presence of this motif (Supplementary Figure S2). These 5΄-capped molecular forms are prioritized as templates for translation, suggesting that the increased noise associated with the presence of G_14_ is created by stochastic processes during scanning that control the access of the ribosomal pre-initiation complex to the reporter start codon.

In conclusion, a combination of experimental and analytical approaches has revealed that inhibitory structures in the 5΄UTR of mRNA can act to promote noise. By measuring both mRNA copy numbers and protein fluorescence intensity in single cells, we have been able to show that the inhibitory elements we have used do not increase noise by modulating transcription or by accelerating mRNA degradation. Since there is a large body of published evidence indicating that 5΄UTR structural elements influence either, or both of, the steps of ribosomal recruitment and scanning (54,59,60), our data suggest that the observed noise generation is associated with changes in translation initiation, most likely affecting the ribosomal scanning process. Our observations contrast with a model in which translation simply acts as an amplifier of transcriptional noise, painting a more complex picture in which structural elements in the 5΄UTR contribute to the generation of (irregular) pulses of gene expression. In the presence of a stable structural element in the 5΄UTR, translational noise adds a layer of additional stochasticity on top of the noise intrinsic to the transcription process and is therefore likely to contribute to the large differences in CV values observed for proteins expressed at similar levels in yeast (24). We have also recently found that restricting the translation efficiency of essential protein synthesis machinery genes in yeast increases their expression noise to atypically high levels (61).

In a wider context, we note that previous work has demonstrated the existence of marked (nongenetic) cell-to-cell variation in the content of mRNA and protein per cell in both lower and higher eukaryotes (1,62). In this study, we have demonstrated the existence of a translational (5΄UTR-mediated) mechanism for generating such noise. This leads to the hypothesis that, for at least a subset of naturally occurring mRNAs, a component of the protein noise in eukaryotic cells is attributable to stochasticity linked to 5΄UTR structure. Our initial bioinformatic analysis reveals correlations between 5΄UTR structure, translation efficiency and noise, thus indicating that it would be informative to conduct a wider study of the relationship between noise and translational inhibition on endogenous mRNAs in lower and higher eukaryotes. In addition, our results will inform synthetic genetic circuitry design for a range of organisms. For example, given that noise suppression can be advantageous in terms of achieving predictable and reliable circuit behavior (63), knowledge of the principles governing translational noise generation will help guide the tuning strategies used to engineer an optimal balance between transcription and translation.

## Supplementary Material

Supplementary Data
