# Supplementary material for: Translation initiation events on structured eukaryotic mRNAs generate gene expression noise
Source: Nucleic Acids Res. 2017 May 17;45(11):6981–92. doi: 10.1093/nar/gkx430 (PMC5499741; doi:10.1093/nar/gkx430)
Supplement: Supplementary Data [file gkx430_Supp.pdf]

Supplementary Data

Strains

	Strain	Integrated plasmid	Plasmid Name
yEGFP	PTC875	*YIp-HK-TEF1p-**L0**-yEGFP_mut3-24xMS2SL-PGK1t*	pNM1-MS2SL
	PTC1006	*YIp-HK-TEF1p-**U**-yEGFP_mut3-24xMS2SL-PGK1t*	pNM1b-MS2SL
	PTC897	*YIp-HK-TEF1p-**M1U**-yEGFP_mut3-24xMS2SL-PGK1t*	pNM2-MS2SL
	PTC899	*YIp-HK-TEF1p-**G10**-yEGFP_mut3-24xMS2SL-PGK1t*	pNM44-MS2SL
	PTC992	*YIp-HK-TEF1p-**M3**-yEGFP_mut3-24xMS2SL-PGK1t*	pNM3c-MS2SL
	PTC901	*YIp-HK-TEF1p-**M3U**-yEGFP_mut3-24xMS2SL-PGK1t*	pNM3-MS2SL
	PTC903	*YIp-HK-TEF1p-**G14**-yEGFP_mut3-24xMS2SL-PGK1t*	pNM45-MS2SL
	PTC825	*YIp-HK-PAB1p-**L0**-yEGFP_mut3-24xMS2SL-PGK1t*	pED27
	PTC905	*YIp-HK-PAB1p-**M1U**-yEGFP_mut3-24xMS2SL-PGK1t*	pED32
	PTC907	*YIp-HK-PAB1p-**G10**-yEGFP_mut3-24xMS2SL-PGK1t*	pED33
	PTC876	*YIp-HK-DCD1p-**L0**-yEGFP_mut3-24xMS2SL-PGK1t*	pNM4-MS2SL
ymNeonGreen	PTC1015	*YIp-HK-TEF1p-**L0**-ymNeonGreen-24xMS2SL-PGK1t*	pED41
	PTC1034	*YIp-HK_TEF1p-**M3wN**-ymNeonGreen-24xMS2SL-PGK1t*	pED51
	PTC1043	*YIp-HK_TEF1p-**M3N**-ymNeonGreen-24xMS2SL-PGK1t*	pED55
	PTC1017	*YIp-HK-TEF1p-**M3UN**-ymNeonGreen-24xMS2SL-PGK1t*	pED42
	PTC1019	*YIp-HK-TEF1p-**G14**-ymNeonGreen-24xMS2SL-PGK1t*	pED43
	PTC1036	*YIp-HK-PAB1p-**L0**-ymNeonGreen-24xMS2SL-PGK1t*	pED52
	PTC1021	*YIp-HK-DCD1p-**L0**-ymNeonGreen-24xMS2SL-PGK1t*	pED44
Dual colour	PTC1023	*YIp-HK-TEF1p-**L0**-mRuby3-ADH1t-TEF1p-**L0**-yEGFP-24xMS2SL-PGK1t*	pED46
	PTC1039	*YIp-HK_TEF1p-**M1Ur**-mRuby3-ADH1t-TEF1p-**M1Ug**-yEGFP_mut3-24xMS2SL-PGK1t*	pED53
	PTC1041	*YIp-HK_TEF1p-**M3r**-mRuby3-ADH1t-TEF1p-**M3g**-yEGFP_mut3-24xMS2SL-PGK1t*	pED54
	PTC1032	*YIp-HK_PAB1p-**L0**-mRuby3-ADH1t-PAB1p-**L0**-yEGFP_mut3-24xMS2SL-PGK1t*	pED50

Selection of inhibitory structural elements for insertion into reporter gene 5'UTRs

A wide range of stem-loop structures were designed based on the observed relationships between predicted folding free energies and inhibitory effect that were described in previous reports [see e.g. Sagliocco F, Vega Laso MR, Zhu D, Tuite MF. McCarthy, JEG, and Brown AJP (1993) The influence of 5' secondary structures upon ribosome binding to mRNA during translation in yeast. *J Biol Chem* 268: 26522-26530; Koloteva N, Müller PP, McCarthy JEG (1997) The position dependence of translational regulation via RNA-RNA and RNA-protein interactions in the 5′-untranslated regions of eukaryotic mRNA is a function of the thermodynamic competence of 40S ribosomes in translational initiation. *J Biol Chem* 272:16531-16539; McCarthy JEG (1998) Posttranscriptional control of gene expression in yeast. *Microbiol Mol Biol Rev* 62: 1492-1553]. The impact of these stem-loop structures was tested using the *LUC* reporter gene (Figure S1). We also compared the effects of two poly(G) elements (G_{10} and G_{14}). A subset of these structural elements was then combined with *yEGFP*, *ymNeonGreen* and *mRuby3* for the noise studies described in this paper. In a number of cases, the juxtaposition of a reporter gene with a stem-loop structure at the mRNA level enabled the formation of unwanted additional base pairs. We accordingly engineered small sequence adjustments in the stem-loop structures in order to maximise the probability that each stem-loop structure would be maintained when in combination with all of the respective reporter gene mRNAs. However, some variation in the predicted stability of each stem-loop type (M1, M3 etc.) expected in combination with the different reporter genes was unavoidable. This did not, however, detract from our key objective, i.e. to explore the trend in gene expression noise for different reporters as the inhibitory potential of 5'UTR structure is increased.

Live-cell imaging of mRNAs

We initially attempted to perform live-cell mRNA quantitations using both tandem MCP/MS2 loops and tandem PCP/PP7 loops in the target mRNA 3'UTRs (compare Wu,B. *et al.* (2012) *Biophys. J.* 102, 2936-2944). However, in our experience, this approach seemed to have a limited capacity to detect more than ten foci and, moreover, the foci were of greatly variable intensity. In addition, a number of questions have arisen in the wider literature about the accuracy of this type of system in

yeast, related to the effects of binding of the bacteriophage coat proteins to the stem-loop structures on the degradation and/or location of the target mRNAs. Such effects can potentially lead to aggregation/accumulation of fragmented mRNAs in p-bodies or other granular structures. References relevant to these points include:

- Garcia,J.F. and Parker,R. (2015) MS2 coat proteins bound to yeast mRNAs block 5' to 3'degradation and trap mRNA decay products: implications for the localization of mRNAs by MS2-MCP system. *RNA*, **8**, 1393-5.
- Haimovich,G., Zabezhinsky,D., Haas,B., Slobodin,B., Purushothaman,P., Fan,L., Levin,J.Z., Nusbaum,C. and Gerst,J.E. (2016) Use of the MS2 aptamer and coat protein for RNA localization in yeast: A response to "MS2 coat proteins bound to yeast mRNAs block 5' to 3' degradation and trap mRNA decay products: implications for the localization of mRNAs by MS2-MCP system". *RNA*, **5**, 660-6.
- Heinrich,S., Sidler,C.L., Azzalin,C.M. and Weis,K. (2017) Stem-loop RNA labeling can affect nuclear and cytoplasmic mRNA processing. *RNA*, **2**, 134-141.

Single molecule Fluorescence *In Situ* Hybridization (smFISH)

We accordingly adopted smFISH as the more reliable and accurate technique for detecting and quantitating full-length mRNAs. The probe sequences used in this study were as follows:

yEGFP_mut3 coding seq

```
aattcttcacctttagacat
aattgggacaacaccagtga
gaccattaacatcaccatct
ccttcaccggagacagaaaa
ttaccgtaagtagcatcacc
actggcaatttaccagtagt
tagtgactaaggttggccat
acattgaacaccataaccga
atatgatctgggtatctcgc
ctggcatggcagacttgaaa
gttctttcttgaacataacc
gtagttaccgtcatctttga
acttgacttcagctctggtc
ttaactaaggtatcaccttc
gacctaaaatgttaccatct
agagttatagttgtattcca
gtcagccatgatgtaaacat
ctttgataccattcttttgt
accatcttcaatgttgtgtc
ttgttgataatggtcagcta
gaccatcaccaattggagta
tggttgtctggtaacaagac
ggcagattgagtggataagt
cttttcgtttggatctttgg
ctaacaagaccatgtggtct
ggtaataccagcagcagtaa
tgtacaattcatccatacca
```

MS2SL

```
attcaattcgccctatagtg
tcgtgctttcttggcaataa
cgtttgaagattcgacctgg
aatactggagcgacgcgtga
accgtaggatctgatgaacc
tcgtgctttcttggcaataa
cgtttgaagattcgacctgg
aatactggagcgacgcgtga
accgtaggatctgatgaacc
tcgtgctttcttggcaataa
cgtttgaagattcgacctgg
aatactggagcgacgcgtga
accgtaggatctgatgaacc
tcgtgctttcttggcaataa
cgtttgaagattcgacctgg
aatactggagcgacgcgtga
accgtaggatctgatgaacc
tcgtgctttcttggcaataa
cgtttgaagattcgacctgg
aatactggagcgacgcgtga
accgtaggatctgatgaacc
tcgtgctttcttggcaataa
gtgatcgtcgtcgtttgaag
tgaaccctggaatactggag
tcgtgctttcttggcaataa
gtgatcgtcgtcgtttgaag
tgaaccctggaatactggag
tcgtgctttcttggcaataa
cgtttgaagattcgacctgg
aatactggagcgacgcgtga
accgtaggatctgatgaacc
tcgtgctttcttggcaataa
cgtttgaagattcgacctgg
aatactggagcgacgcgtga
accgtaggatctgatgaacc
tcgtgctttcttggcaataa
cgtttgaagattcgacctgg
aatactggagcgacgcgtga
accgtaggatctgatgaacc
tcgtgctttcttggcaataa
cgtttgaagattcgacctgg
aatactggagcgacgcgtga
accgtaggatctgatgaacc
tcgtgctttcttggcaataa
cgtttgaagattcgacctgg
aatactggagcgacgcgtga
ttcgcgagatctgatgaacc
gtcctgcaggtttaaacgaa
```

We have followed previously published guidance on testing different concentrations of smFISH probes for each transcript to be detected (see, for example, Raj, A. and Tyagi, S. (2010) Detection of individual endogenous RNA transcripts in situ using multiple singly labeled probes. *Methods in Enzymology*, **472**, 365-386).

This allowed us to optimise the signal-to-noise ratio for measurements of mRNAs of different

abundance. We found out that using a low concentration of FISH probes for low expression transcripts and higher concentration for high expression transcripts gave the best results.

Image acquisition and analysis:

FISH images were acquired on a Deltavision Elite, an epifluorescence inverted widefield microscope, equipped with a 60x PlanApo objective, 1.42 numerical aperture using 1.515 refractive index oil and a CCD camera. The temperature of the incubation chamber was set at 25°C for optimal functioning of the glucose oxidase and catalase contained in the GLOX buffer. Cells were imaged in three dimensions with z-steps of 200 nm and a total z-stack of 5.8 µm. Pixel size in x and y was 107 nm. A four-colour image was acquired for each field of view using CY5 (excitation 632/22 nm, emission 676/34 nm), TRITC (excitation 542/27 nm, emission 594/45 nm), FITC (excitation 475/28 nm, emission 532/36 nm) and DAPI (excitation 390/18 nm, emission 435/48 nm) filter sets in this order.

Cell segmentation and image analysis were performed using FISH-quant [Mueller F, Senecal A, Tantale K, Marie-Nelly H, Ly N, Collin O, Basyuk E,Bertrand E, Darzacq X, Zimmer C. FISH-quant: automatic counting of transcripts in 3D FISH images. *Nat Methods*. 10 (2013) 277-8; Tsanov N et al (2016) smiFISH and FISH-quant – a flexible single RNA detection approach with super-resolution capability. *Nucleic Acids Res* 44: e165]. For each FISH experiment, the background strain PTC830 (not expressing any reporter) was imaged alongside the sample strain as a negative control in order to determine the optimal intensity threshold to use to detect all real spots while keeping the false positive detection rate as low as possible (below 2.5% for all experiments).

Preparation of cells for flow cytometry

Two days prior to an experiment, single colonies from each of the strains were picked and grown overnight in YNB (plus amino acids, 2% glucose) to saturation with shaking at 30°C. The following morning, cells were diluted to give an optical density at 600 nm (OD_{600}) of ~0.1 and incubated further to an OD_{600} of about ~0.8 to 1.0. The cultures were then diluted again to an OD_{600} of ~0.0001 and allowed to grow overnight. On the morning of the experiments, cultures were handled

in batches of 3 or 4: the cultures were diluted to an OD_{600} ~0.2, and these diluted cultures were grown to mid-log phase (OD_{600} ~0.4 to 0.5) with shaking at 30°C. This procedure allowed us to maintain the cultures in the exponential growth phase right up to the time of measurement.

100µl of mid-log phase culture from each strain were transferred to individual plastic tubes. The cells were subjected to low-power sonication at 50-60Hz for approximately 10 seconds in order to separate any cell aggregates. After sonication, the cell suspensions were further diluted by adding 900 µL of PBS to each tube prior to the flow cytometry measurements. To minimize variation due to delays between sample delivery and measurements, the individual strains were prepared for flow cytometry at intervals of 20 minutes.

Noise data obtained with different reporters

In this study, we used three different reporter genes. Comparison of the noise estimates obtained with yEGFP and ymNeonGreen (Table 1, Figure 4) reveals that the absolute estimates of noise for equivalent constructs were not identical. This is likely to be due primarily to two factors: 1. Differences in the 5'-proximal sequences of the respective reporter genes meant that we could not use identical 5'UTR sequences for both of the genes, and also that the secondary structures around the start codons were non-identical and possibly followed different folding/unfolding kinetics; 2. The folding times and degradation rates for the encoded fluorescent proteins are probably non-identical, which in turn means that the overall lifetimes of the fluorescently active species are different. Despite these differences, the trends of translation rate vs measured CV for the two genes were remarkably consistent.

We constructed genomic dual expression constructs using the *yEGFP* and *mRuby3* reporter genes (Figure 5; Table S2). The mean fluorescence data and CV values for yEGFP in the directly comparable single-reporter (Table 1) and dual-reporter (Table S2; Figure 5) constructs reveal strong consistency. However, changes also had to be made in the 5'UTRs when these were paired with the *mRuby3* reading frame, and these changes, for the reasons mentioned above, could have been at least partially responsible for the differences in absolute noise levels observed with the respective reporters.

R code for analysis of two-colour flow cytometry data

```
###This R code is written to calculate statistics for each flow cytometry data
file in FCS 3.0
rm(list=ls(all=TRUE))
graphics.off()
gc()
library(flowCore)
library(flowViz)
library(flowDensity)
#load the fcs 3.0 data file
fs <- read.flowSet(pattern='.fcs')
#plot(fs[[1]],c('FSC-A','SSC-
A'),main=sampleNames(fs)[1],ylim=c(0,200000),smooth=FALSE)
time.value <- exprs(fs[[1]])[,'Time']
time.post <- which(exprs(fs[[1]])[,'Time'] > 100 & exprs(fs[[1]])[,'Time'] <
(max(time.value)-20))
fs.time <- fs[[1]][time.post]
#plot(fs.time,c('FSC-A','SSC-
A'),main=sampleNames(fs)[1],ylim=c(0,200000),smooth=FALSE)
fsca.value <- exprs(fs.time)[,'FSC-A']
fsca.post <- which(exprs(fs.time)[,'FSC-A'] > 40000 & exprs(fs.time)[,'FSC-A'] <
100000)
ssca.value <- exprs(fs.time)[,'SSC-A']
ssca.post <- which(exprs(fs.time)[,'SSC-A'] > 10000 & exprs(fs.time)[,'SSC-A'] <
90000)
combine.post <- intersect(fsca.post,ssca.post)
fs.select <- fs.time[combine.post]
plot(fs.select,c('FSC-A','SSC-
A'),main=sampleNames(fs)[1],xlim=c(0,200000),ylim=c(0,200000),smooth=TRUE)
interval <- 1500
i <- seq(from=0, to=200000-interval, by=interval)
map <- matrix(0.6,length(i),length(i))
maptab <- matrix(0.6,length(i)^2,7,dimnames=list(c(),
c('m','fsc.No','n','ssc.No','cmb.No','x','y')))
x <- 0
max.cells <- 0
j <- 1
for (m in 1:length(i)){
  cat('  m=',m)
  cat('  x=',x)
  fsca.post <- which(exprs(fs.select)[,'FSC-A'] > x & exprs(fs.select)[,'FSC-A']
< x+interval)
  cat('  fsc=',length(fsca.post))
  cat('\n')
  y <- 0
  for (n in 1:length(i)){
    cat('  n=',n)
    cat('  y=',y)
    ssca.post <- which(exprs(fs.select)[,'SSC-A'] > y & exprs(fs.select)[,'SSC-
A'] < y+interval)
    cat('  ssc=',length(ssca.post))
    cat('\n')
    combine.post <- intersect(fsca.post,ssca.post)
    map[length(i)-n+1,m] <- length(combine.post)
    cat('\n')
    cat('\f')
    if(map[length(i)-n+1,m] >= max.cells){
      maptab[j,1] <- m
      maptab[j,2] <- length(fsca.post)
      maptab[j,3] <- n
      maptab[j,4] <- length(ssca.post)
      maptab[j,5] <- length(combine.post)
      maptab[j,6] <- x
```

```
      maptab[j,7] <- y
      max.cells <- map[length(i)-n+1,m]
      j <- j+1
    }
    y <- y+interval
  }
  x <- x+interval
}
#write.csv(map,file = 'map.csv',row.names = FALSE)
#write.csv(maptab,file = 'maptab.csv',row.names = FALSE)
max.position <- which(maptab[,5] == max(maptab[,5]))
if (length(max.position) > 1){
  text(100000,100000,'ERROR!\nMAX > 1\nReset interval',col='red',cex=2)
  maptab[max.position[1],]
  maptab[max.position[2],]
} else {
  maptab[max.position[1],]
  center.x <- maptab[max.position[1],6]+interval/2
  center.y <- maptab[max.position[1],7]+interval/2
  points(center.x,center.y,col='red',cex=3,pch='.')
}
cell.list <- matrix(1,nrow(fs.select),14,dimnames=list(c(), c('No.k',
'distance','GFP','nrGFP-med','RFP','nrRFP-med','FSC-axis','SSC-axis','(nrG-
nrR)^2','nrG*nrR','nrG^2+nrR^2','center.x','center.y','interval')))
FSC <- seq(from = 1, to = nrow(fs.select), by = 1)
SSC <- seq(from = 1, to = nrow(fs.select), by = 1)
GFP <- seq(from = 1, to = nrow(fs.select), by = 1)
RFP <- seq(from = 1, to = nrow(fs.select), by = 1)
r <- seq(from = 1, to = nrow(fs.select), by = 1)
med.GFP <- median(exprs(fs.select)[,'B488-530/30-A'])
med.RFP <- median(exprs(fs.select)[,'YG561-610/20-A'])
for (k in 1:nrow(fs.select)){
  FSC[k] <- exprs(fs.select)[,'FSC-A'][k]
  SSC[k] <- exprs(fs.select)[,'SSC-A'][k]
  GFP[k] <- exprs(fs.select)[,'B488-530/30-A'][k]
  RFP[k] <- exprs(fs.select)[,'YG561-610/20-A'][k]
  r[k] <- sqrt((FSC[k]-center.x)^2+(SSC[k]-center.y)^2)
  cell.list[k,1] <- k
  cell.list[k,2] <- r[k]
  cell.list[k,3] <- GFP[k]
  cell.list[k,4] <- GFP[k]/med.GFP
  cell.list[k,5] <- RFP[k]
  cell.list[k,6] <- RFP[k]/med.RFP
  cell.list[k,7] <- FSC[k]
  cell.list[k,8] <- SSC[k]
  nrGFP <- cell.list[k,4]
  nrRFP <- cell.list[k,6]
  cell.list[k,9] <- (nrGFP-nrRFP)^2
  cell.list[k,10] <- nrGFP*nrRFP
  cell.list[k,11] <- nrGFP^2+nrRFP^2
  cell.list[k,12] <- center.x
  cell.list[k,13] <- center.y
  cell.list[k,14] <- interval
  cat(floor(k/nrow(fs.select)*100),'%','|||')
  if ((k)%%2==0){
    cat('\f')
  }
}
#write.csv(cell.list,file = 'cell-list.csv',row.names = FALSE)
decrease.post<- order(cell.list[,2],decreasing=FALSE)
order2col <- cell.list[decrease.post,]
#write.csv(order2col,file = 'order2col.csv',row.names = FALSE)
trimGFP.max = 0.5
trimGFP.min = 0.5
```

```
trimRFP.max = 0.5
trimRFP.min = 0.5
orderGFP.post <- order(order2col[,3],decreasing=FALSE)
trimGFP.post <-
orderGFP.post[((nrow(order2col)*(trimGFP.min/100))+1):(nrow(order2col)*(1-
trimGFP.max/100))]
#-order2col
orderRFP.post <- order(order2col[,5],decreasing=FALSE)
trimRFP.post <-
orderRFP.post[((nrow(order2col)*(trimRFP.min/100))+1):(nrow(order2col)*(1-
trimRFP.max/100))]
combine.post2 <- intersect(trimGFP.post,trimRFP.post)
trim.percentage <- (nrow(order2col)-length(combine.post2))/nrow(order2col)*100
trim2col <- order2col[combine.post2,]
plot(order2col[,4],order2col[,6],pch='.',xlim=c(0,4),ylim=c(0,4),col='red',xlab=
'Normalised GFP (a.u.)',ylab='Normalised RFP (a.u.)',las=1)
points(trim2col[,4],trim2col[,6],pch='.',xlim=c(0,4),ylim=c(0,4),col='blue')
text(3.6,3.6,sprintf("%1.1f%%", -trim.percentage),col='blue',cex=1.5)
distance.post<- order(trim2col[,2],decreasing=FALSE)
trim2col <- trim2col[distance.post,]
#=trim2col
write.csv(trim2col,file = 'trim2col.csv',row.names = FALSE)
#-trim2col
table2col <- matrix(1,(nrow(trim2col)-1),14,dimnames=list(c(), c('cell number',
'max distance','GFP-mean','GFP-cv%','GFP-Fano','RFP-mean','RFP-cv%','RFP-
Fano','intrinsic','extrinsic','total','Fano.int','Fano.ext','Fano.total')))
for (t in 1:(nrow(trim2col)-1)){
  GFPcv <- (sd(trim2col[1:(t+1),3])/mean(trim2col[1:(t+1),3]))*100
  GFPfano <- (sd(trim2col[1:(t+1),3])^2)/mean(trim2col[1:(t+1),3])
  RFPcv <- (sd(trim2col[1:(t+1),5])/mean(trim2col[1:(t+1),5]))*100
  RFPfano <- (sd(trim2col[1:(t+1),5])^2)/mean(trim2col[1:(t+1),5])
  table2col[t,1] <- t+1
  table2col[t,2] <- max(trim2col[1:(t+1),2])
  table2col[t,3] <- mean(trim2col[1:(t+1),3])
  table2col[t,4] <- GFPcv
  table2col[t,5] <- GFPfano
  table2col[t,6] <- mean(trim2col[1:(t+1),5])
  table2col[t,7] <- RFPcv
  table2col[t,8] <- RFPfano
  table2col[t,9] <-
sqrt(mean(trim2col[1:(t+1),9])/(2*mean(trim2col[1:(t+1),4])*mean(trim2col[1:(t+1
),6])))
  table2col[t,10] <- sqrt((mean(trim2col[1:(t+1),10])-
mean(trim2col[1:(t+1),4])*mean(trim2col[1:(t+1),6]))/mean(trim2col[1:(t+1),4])*m
ean(trim2col[1:(t+1),6]))
  table2col[t,11] <- sqrt((mean(trim2col[1:(t+1),11])-
2*mean(trim2col[1:(t+1),4])*mean(trim2col[1:(t+1),6]))/(2*mean(trim2col[1:(t+1),
4])*mean(trim2col[1:(t+1),6])))
  table2col[t,12] <-
mean(trim2col[1:(t+1),9])/(2*sqrt(mean(trim2col[1:(t+1),4])*mean(trim2col[1:(t+1
),6])))
  table2col[t,13] <- (mean(trim2col[1:(t+1),10])-
mean(trim2col[1:(t+1),4])*mean(trim2col[1:(t+1),6]))/sqrt(mean(trim2col[1:(t+1),
4])*mean(trim2col[1:(t+1),6]))
  table2col[t,14] <-
(table2col[t,11])^2*(sqrt(mean(trim2col[1:(t+1),4])*mean(trim2col[1:(t+1),6])))
  cat(floor(t/(nrow(trim2col)-1)*100),'%','|||')
  if ((t+1)%%2==0){
    cat('\f')
  }
}
write.csv(table2col,file = 'table2col.csv',row.names = FALSE)
##
pdf("plots2col.pdf",width=8, height=8)
```

```
#
plot(fs[[1]],c('FSC-A','SSC-
A'),main=sampleNames(fs)[1],ylim=c(0,200000),smooth=FALSE)
#
plot(fs.select,c('FSC-A','SSC-
A'),main=sampleNames(fs)[1],xlim=c(0,200000),ylim=c(0,200000),smooth=TRUE)
points(center.x,center.y,col='red',cex=3,pch='.')
#
plot(order2col[,4],order2col[,6],main=sampleNames(fs)[1],pch='.',xlim=c(0,4),yli
m=c(0,4),col='red',xlab='Normalised GFP (a.u.)',ylab='Normalised RFP
(a.u.)',las=1)
points(trim2col[,4],trim2col[,6],pch='.',xlim=c(0,4),ylim=c(0,4),col='blue')
text(3.6,3.6,sprintf("%1.1f%%", -trim.percentage),col='blue',cex=1.5)
#
plot(table2col[,2],table2col[,4],main=sampleNames(fs)[1],col='green',pch='.',cex
=3,xlab='radius
(a.u.)',ylab='CV %',xlim=c(1000,100000),ylim=c(0,50),log='x',xaxt='n',las=1)
axis(1,at=c(1000,2000,3000,4000,5000,6000,7000,8000,9000,10000,100000),
labels=c('1k','','','','5k','','','','','10k','100k'), cex.axis=0.8)
axis(1,at=c(3000,5000,10000),labels=c('3k','5k','10k'), cex.axis=0.8)
axis(1,at=c(seq(20000,100000,10000)),labels=FALSE)
points(table2col[,2],table2col[,7],col='red',pch='.',cex=3,xlab='radius
(a.u.)',ylab='CV %',xlim=c(1000,100000),ylim=c(0,50),log='x')
legend("topright",legend=c('GFP','RFP'),col=c('green','red'),pch=20,lwd=2,cex=1.
5,bty='n')
#
plot(table2col[,2],table2col[,9]*100,main=sampleNames(fs)[1],col='purple',pch='.
',cex=3,xlab='radius
(a.u.)',ylab='CV %',xlim=c(1000,100000),ylim=c(0,50),log='x',xaxt='n',las=1)
axis(1,at=c(1000,2000,3000,4000,5000,6000,7000,8000,9000,10000,100000),
labels=c('1k','','','','5k','','','','','10k','100k'), cex.axis=0.8)
axis(1,at=c(3000,5000,10000),labels=c('3k','5k','10k'), cex.axis=0.8)
axis(1,at=c(seq(20000,100000,10000)),labels=FALSE)
points(table2col[,2],table2col[,10]*100,col='blue',pch='.',cex=3,xlab='radius
(a.u.)',ylab='CV %',xlim=c(1000,100000),ylim=c(0,50),log='x')
points(table2col[,2],table2col[,11]*100,col='black',pch='.',cex=3,xlab='radius
(a.u.)',ylab='CV %',xlim=c(1000,100000),ylim=c(0,50),log='x')
legend("topleft",legend=c('Intrinsic','Extrinsic','Total'),col=c('purple','blue'
,'black'),pch=20,lwd=2,cex=1.5,bty='n')
#
plot(table2col[,2],table2col[,9]*100,main=sampleNames(fs)[1],col='purple',pch='.
',cex=3,xlab='radius
(a.u.)',ylab='CV %',xlim=c(1000,100000),ylim=c(0,50),log='x',xaxt='n',las=1)
axis(1,at=c(1000,2000,3000,4000,5000,6000,7000,8000,9000,10000,100000),
labels=c('1k','','','','5k','','','','','10k','100k'), cex.axis=0.8)
axis(1,at=c(3000,5000,10000),labels=c('3k','5k','10k'), cex.axis=0.8)
axis(1,at=c(seq(20000,100000,10000)),labels=FALSE)
points(table2col[,2],table2col[,10]*100,col='blue',pch='.',cex=3,xlab='radius
(a.u.)',ylab='CV %',xlim=c(1000,100000),ylim=c(0,50),log='x')
points(table2col[,2],table2col[,11]*100,col='black',pch='.',cex=3,xlab='radius
(a.u.)',ylab='CV %',xlim=c(1000,100000),ylim=c(0,50),log='x')
legend("topleft",legend=c('Intrinsic','Extrinsic','Total'),col=c('purple','blue'
,'black'),pch=20,lwd=2,cex=1.5,bty='n')
points(table2col[,2],table2col[,4],col='green',pch='.',cex=3,xlab='radius
(a.u.)',ylab='CV %',xlim=c(1000,100000),ylim=c(0,50),log='x',xaxt='n',las=1)
points(table2col[,2],table2col[,7],col='red',pch='.',cex=3,xlab='radius
(a.u.)',ylab='CV %',xlim=c(1000,100000),ylim=c(0,50),log='x')
legend("topright",legend=c('GFP','RFP'),col=c('green','red'),pch=20,lwd=2,cex=1.
5,bty='n')
dev.off()
```

Computational model

The computational model depicted in Figure 7 was developed based on the model of Kaern et al. (ref 15) which was used to study transcriptional intrinsic noise. The model represents a gene that can be in two states, D_0 and D_1, where the former is transcribed at a low basal rate, and the latter is an activated state with a higher rate of transcription. The gene exists in two copies which can be in either state; the transition from D_0 to D_1 is through an implicit binding of a transcription factor with association constant $K_{eq} = k_{on}/k_{off}$. Transcription of the gene generates an mRNA species R_0 at rates α_0 and α_1 where $\alpha_1 = 10 \times \alpha_0$. R_0 is degraded in a first-order process with rate constant γ_R. R_0 supports translation producing a protein P at a translation rate $\beta \times [R_0]$. The protein is degraded in a first-order process with rate constant γ_P. The part of this model that differs from Kaern et al. (15) is that there is a process of folding of the mRNA into a species R_f, which is not able to be used for translation. The transition between R_0 and R_0 happens through a first-order reversible reaction with rate constants k_{fo} and k_{un}. The stability of the folded mRNA is characterized by equilibrium constant $K_{eq}F= k_{fo}/k_{un}$.

The model is then simulated using one of Gillespie's stochastic simulation algorithms (the direct method, ref. 29) in the software COPASI (30). Simulations are carried out from initial conditions of a stable steady state (calculated using the equivalent ODE system). Noise levels are assessed by simulating 500 time courses of 1000 minutes each with 100 samplings at regular 10-minute intervals. The coefficient of variation (CV) is then calculated from the 50,000 sampled values (500x100). In Figure 7 we investigated the dependency of the protein noise level (CV) on the rate of folding k_{fo} and the folding stability $K_{eq}F$ through a parameter scan. Each point at the intersections of the 2D surface were calculated with the procedure above for a fixed pair of values of k_{fo} and $K_{eq}F$ (*i.e.* for each point 500 simulations were carried out). Figure 7 shows that noise increases with the stability of the folded mRNA (high $K_{eq}F$ values) and at high rate of folding (k_{fo}). For comparison, in Figure S6 we plot the dependency of the protein noise level on the rate of gene activation k_{on} and the dissociation constant of the transcription factor K_d using the same procedure as for Fig. 7. Noise is higher at an intermediate rate of transition between gene states (k_{on} of about 0.5 min^{-1}) and at low promoter binding affinity (K_{eq} of 10^{-3}).

Both SBML and COPASI versions of the model have been added as zip files. The SBML version is portable and can be used by any software capable of reading SBML. The COPASI version is only for the open source software COPASI but includes both the model and the simulation specifications.

Analysis of genome-wide expression and noise data

The study of Sen *et al* (49) was designed to identify mRNAs with complex 5'UTRs whose translation is Ded1-dependent (it is thought that this is because this RNA helicase is required to facilitate scanning through the 5'UTR structure). We have extracted the noise data for those genes in the Sen *et al* study (49) that are also represented in the dataset of Stewart-Ornstein *et al* (50; group 1). Analysis of these genes reveals a statistically significant* correlation between translational dependence on Ded1 and the (primarily intrinsic) noise for the genes. The Table below also summarises the following observations: the mean intrinsic noise for a subset (group 2) of genes identified by Sen *et al* as having strong dependence on Ded1 is significantly greater than for group 1 overall; the mean intrinsic noise for the subset of group 2 that lacks a TATA-box (group 3; 36 genes) is greater than that of group 2 as a whole. For comparison, we show the equivalent noise data for all genes common to both studies that have a TATA-box (group 4) and all genes common to both studies that lack a TATA-box (group 5).

**Selection of genes**	**CV† total**	**CV† intrin.**	**CV† extrin.**
**1:** All genes common to both studies (438)	0.22	0.17	0.13
**2:** 45 genes from most Ded1-dependent group (Sen et al 2015)	0.24	0.20	0.12
**3:** Members (36) of group 2 that have no TATA-box	0.25	0.21	0.11
**4:** All TATA-plus genes (128)	0.26	0.19	0.16
**5:** All TATA-less genes (308)	0.20	0.16	0.11

*The p value = 0.0015 for the hypothesis that the observed correlation between intrinsic noise and reduced translation efficiency in the absence of Ded1 could be due to random sampling.
†Coefficient of variation (total, intrinsic and extrinsic, respectively†).

Table S1
Predicted folding energies

5'UTR construct	ΔG folding (kcal mol^{-1})*
**MIUg**	**- 2.6**
**M3g**	**- 17.7**
**M3Ug**	**- 17.5**
**M3n**	**- 18.6**
**M3Un**	**- 18.4**
**M3Wn**	**- 12.4**
**M1Ur**	**- 3.5**
**M3r**	**-18.6**

The free energy values for the stability of the respective stem-loops have been estimated using published software [Zuker M (2003) Mfold web server for nucleic acid folding and hybridization prediction. *Nucleic Acids Res* 31: 3406-15]. The relationship between predicted stability and degree of translational inhibition is not strictly proportional, since the nucleotide sequence can also influence scanning.

Table S2. mRuby3 / yEGFP ratio experiments

	**P_{TEF1}(L0)**		**P_{TEF1}(M1U)**		**P_{TEF1}(M3)**		**P_{PAB1}(L0)**	
r = 4000	Average	Std dev	Average	Std dev	Average	Std dev	Average	Std dev
Cell number	1004	105	905	136	1002	81	932	78
EGFP-mean	1622	25	419	11	240	5	263	7
EGFP-CV%	12.1	0.6	13.4	0.5	15.5	0.7	14.6	0.7
mRuby3-mean	5580	274	2459	102	1102	92	967	37
mRuby3-CV%	12.2	0.7	13.1	0.9	16.3	0.3	16.5	0.8
intrinsic	0.078	0.006	0.099	0.006	0.125	0.006	0.128	0.007
extrinsic	0.070	0.006	0.065	0.007	0.075	0.008	0.066	0.008
total	0.122	0.006	0.133	0.007	0.159	0.005	0.156	0.007

These data derive from the experiments presented in Fig. 5, and are summarised in the bar graph in Fig. 5E.

A

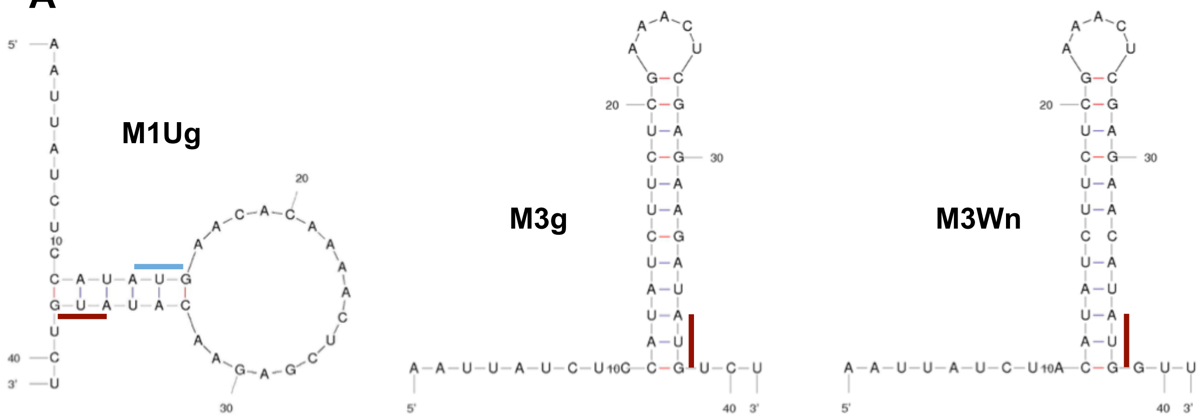

B

	BamH1
**L0**	GGATCCAATTATCTACTTAAGAACACAAAACTCGAGAACAT**ATG**
**M1Ug**	GGATCCAATTATCTCCATATGAACACAAAACTCGAGAACAT**ATG**
**M1Ur**	GGATCCAATTATCTACATATGAACACAAAACTCGAGAACAT**ATG**
**M3Wn**	GGATCCAATTATCTACATATCTTCTCGAAACTCGAGAACAT**ATG**
**M3g**	GGATCCAATTATCTCCATATCTTCTCGAAACTCGAGAAGAT**ATG**
**M3n/M3r**	GGATCCAATTATCTACATATCTTCTCGAAACTCGAGAAGAT**ATG**
**M3Ug**	GGATCCAATTATCTCCATATGTTCTCGAAACTCGAGAACAT**ATG**
**M3Un**	GGATCCAATTATCTACATATGTTCTCGAAACTCGAGAACAT**ATG**

C

Reporter mRNA sequences

LUC	**AUG**GAAGACGCCAAAAACAUAAAGAAAGGCCCGGCGCCAUUCUAUCCACUAGAGGAU
yEGFP	**AUG**UCUAAAGGTGAAGAAUUAUUCACUGGUGUUGUCCCAAUUUUGGUUGAAUUAGAU
ymNG	**AUG**GUUUCTAAGGGUGAAGAAGACAACAUGGCUUCUUUGCCAGCUACUCACGAAUUG
mRuby3	**AUG**GUGUCCAAAGGAGAGGAGUUAAUCAAGGAAAACAUGAGAAUGAAAGUUGUCAUG

Figure S1. 5'UTR and reporter gene sequences used in this study. **A:** Three key examples of stem-loop structures that impose different degrees of inhibition of translation initiation in the appropriate range. The red bar indicates the position of the start codon of the reporter gene; the blue bar shows the position of the uORF AUG. The folding predictions were made using established software (Zuker M (2003) Mfold web server for nucleic acid folding and hybridization prediction. *Nucleic Acids Res* 31: 3406-15). **B:** The DNA sequences of the respective 5'UTRs, highlighting the position of the BamH1 site (located at the major transcr-iptional start site). In some cases, single site mutations were introduced to prevent the fomation of unwanted additional base pairs that would otherwise arise when the 5'UTRs were combined with the respective reporter gene sequences. The resulting variants of the 5'UTRs are indicated as follows: *yEGFP* (g); *ymNG* (n); *mRuby3* (r). The underlined nucleotides are those engaged in base pairing (compare panel **A**). U indicates the presence of an upstream start codon that initiates an overlapping uORF. **C:** The reporter mRNA sequences (first 19 codons), starting with the start codon (in red). The stop codons (UAG and UAA) highlighted in purple signify the translation termination points for +1 short reading frames initiated by upstream AUGs in the M1Ug/r and M3Ug constructs. The *LUC* gene was used in the initial stage of searching for structures (guided by previously published work) that provide the required range of translation inhibition; noise measurements were only performed using the fluorescent reporter protein constructs.

D

G10 GGATCCAATTATCTACTTAAGGGGGGGGGGCTCGAGAACAT**ATG**

G14 GGATCCAATTATCTACTTAAGGGGGGGGGGGGGGAGAACAT**ATG**

E

uORF → yEGFP →

U CTTCTTGTTCATTAGAAAGGATCCAATTATCTACTT**ATG**AACTCGAAACTCGAGAACAT**ATG**
TCTAAAGGTGAAGAATTATTCACTGGTGTTGTCCCAATTTTGGTTGAAT**TAG**ATGGTGATGT

uORF stop

Figure S1. D: The 5'UTR sequences carrying the G_{10} and G_{14} elements.
E: The U 5'UTR/*yEGFP* sequence indicating the location of the uORF.

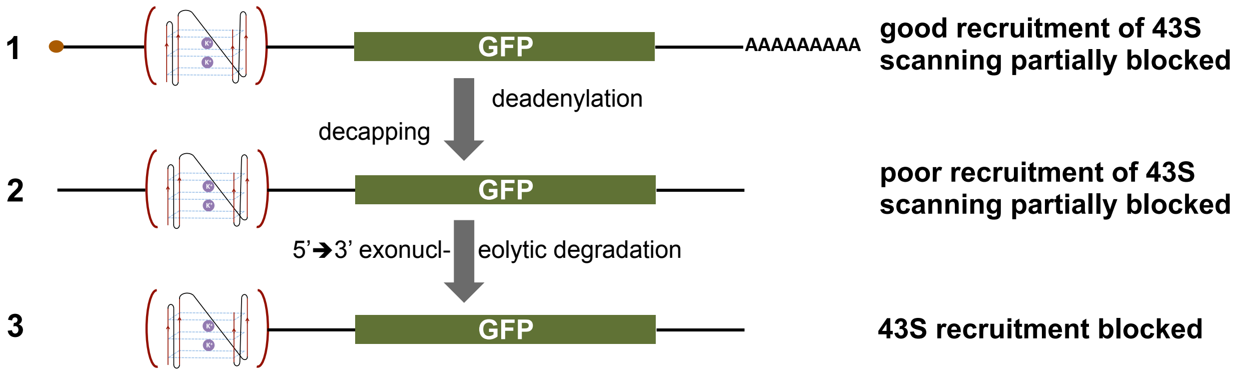

Figure S2. mRNA degradation in the presence of poly(G). The key species detected by smFISH are illustrated. In the predominant decay pathway, deadenylation of intact mRNA (1) promotes decapping, generating a species (2) that is poorly recruited to 40S ribosomal subunits. Where decapping occurs before deadenylation is complete, this also results in multiple species (not explicitly shown here) that are poorly recruited to 40S ribosomal subunits. The (folded) poly(G) structure inhibits scanning in all species of mRNA shown. It also inhibits 5' - 3' exonucleolytic degradation (3), thus stabilising decapped mRNAs. Thus the *additional* mRNA molecules identified by smFISH are poorly translatable or non-translatable species that play at most a minor role in gene expression; these species cannot therefore contribute substantially to the observed changes in protein-level noise.

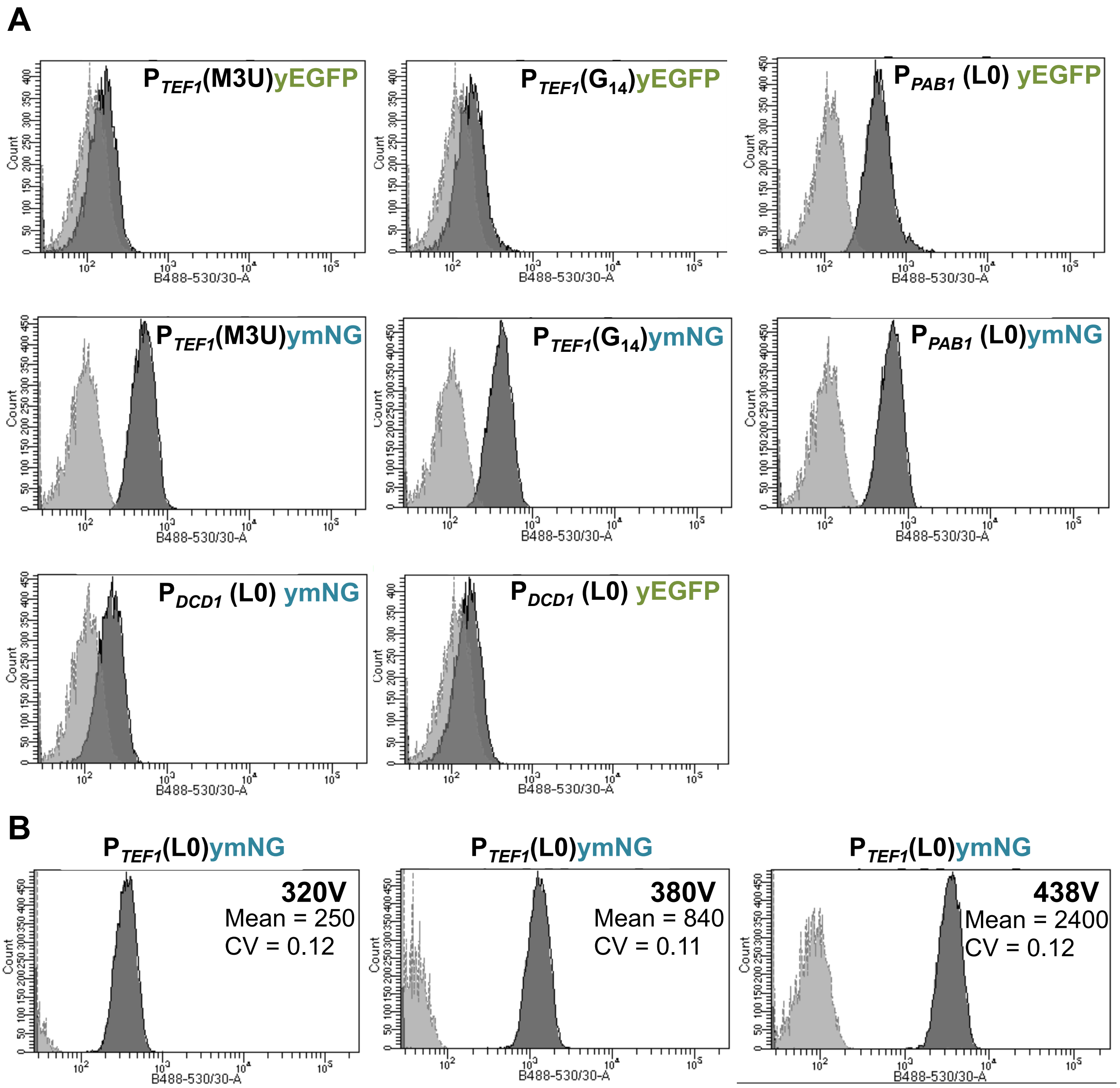

Figure S3 Addressing technical questions related to flow cytometry. **A:** Illustrations of how the greater intensity of ymNeonGreen (ymNG) fluorescence (dark grey signal distribution) eliminates/ minimises overlap with host strain autofluorescence (light grey peak, measured using the host strain lacking any fluorescent reporter constructs) for two of the most strongly inhibited constructs. **B:** Control demonstrating that reducing the detected mean intensity of fluorescence (by reducing the photomultiplier voltages) does not change the observed noise values significantly. Flow cytometry was performed on a strain carrying the genomic P_{TEF1} (L0) ymNG construct. Each plot of fluorescence intensity vs cell number features the mean fluorescence intensity and CV value for the subset of cells identified by gating (gating radius set at 4000; see SI Appendix). In further experiments (not shown here), we found that measurements (at 438V) of the strain bearing the genomic P_{TEF1} (G_{14}) ymNG construct also manifested a mean fluorescence value of 250 [compare P_{TEF1}(L0)ymNG (at 320V) above], but a CV value of 0.154. These data therefore demonstrate that the flow cytometry instrument does not artefactually boost noise at lower fluorescence intensities.

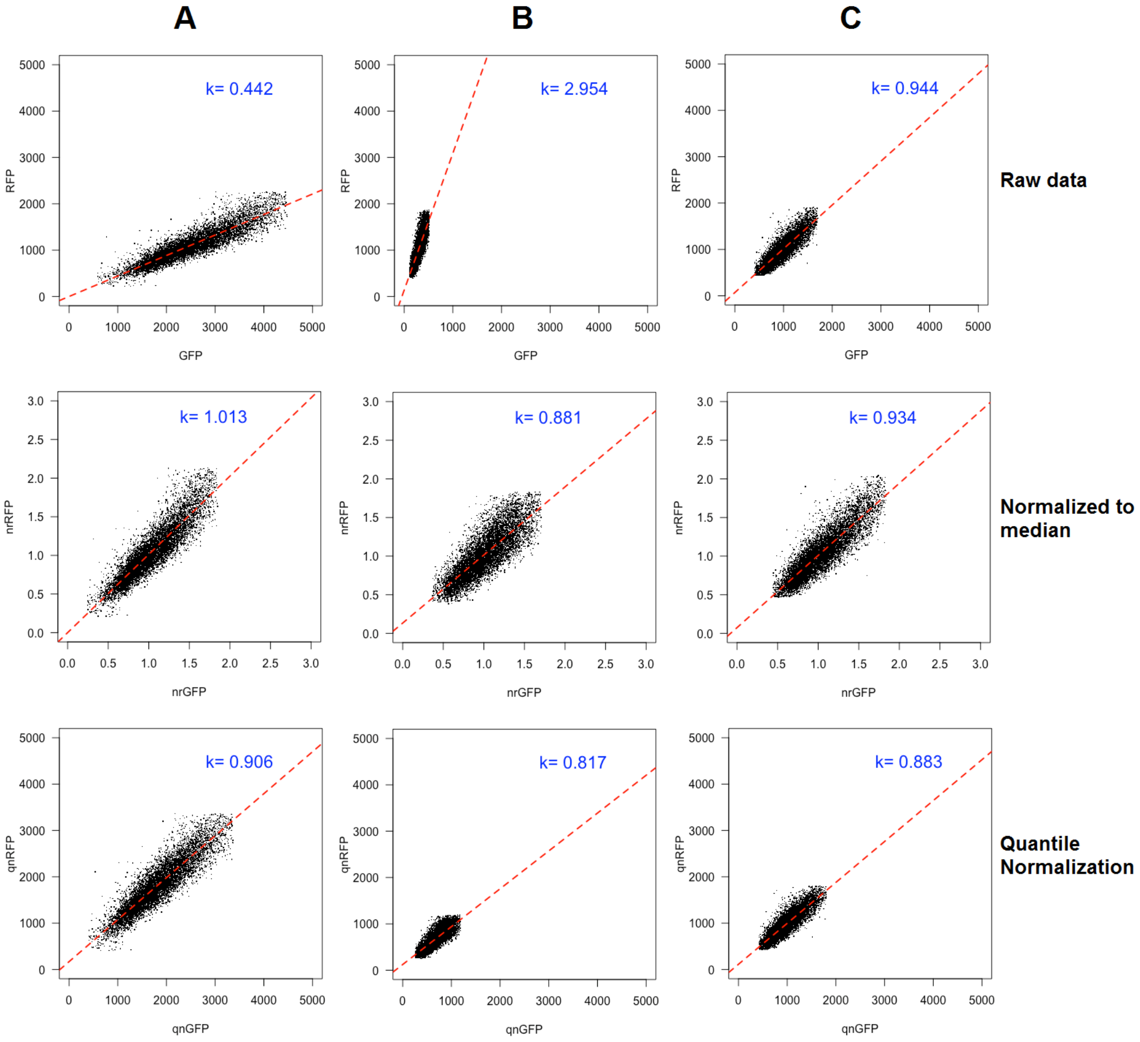

Figure S4. Normalisation of flow cytometry. We compared two alternative procedures for normalisation of dual reporter data. We illustrate here three different situations, in which the raw data show the following relationships: GFP > RFP (*A*), GFP < RFP (*B*), and GFP ≈ RFP (*C*). The slope (k value) of each regression line was calculated for the raw data (GFP vs RFP), after normalization to the median (nrGFP vs nrRFP; Newman *et al*, 2006), and after quantile normalization (qrGFP vs qrRFP; Fu & Pachter, 2016), respectively. Informed by such k values, we chose to adopt the median normalisation method described by Newman *et al (2006)* as our standard procedure.

Newman JR, Ghaemmaghami S, Ihmels J, Breslow DK, Noble M, *et al* (2006) Single-cell proteomic analysis of *S. cerevisiae* reveals the architecture of biological noise. *Nature* 441: 840–846.

Fu AQ, Pachter L (2016) Estimating intrinsic and extrinsic noise from single-cell gene expression measurements. *Stat Appl Genet Mol Biol* 15: 447-471.

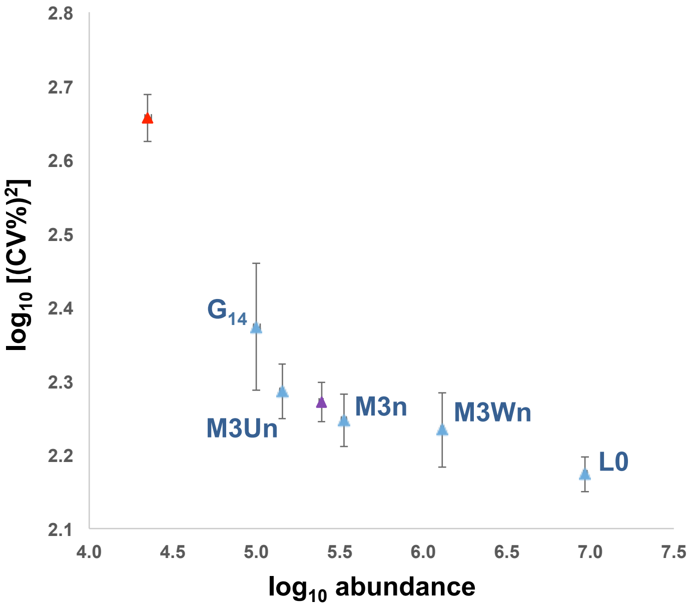

Figure S5. Plot of noise strength vs mean fluorescence intensity using the same data shown in Fig. 6B but including P_{DCD1}(L0; data point in red). The point in purple is P_{PAB1}(L0). Note that the estimate of noise for P_{DCD1}(L0) is affected by overlap with autofluorescence (Fig. S3). Standard deviation values are represented by error bars.

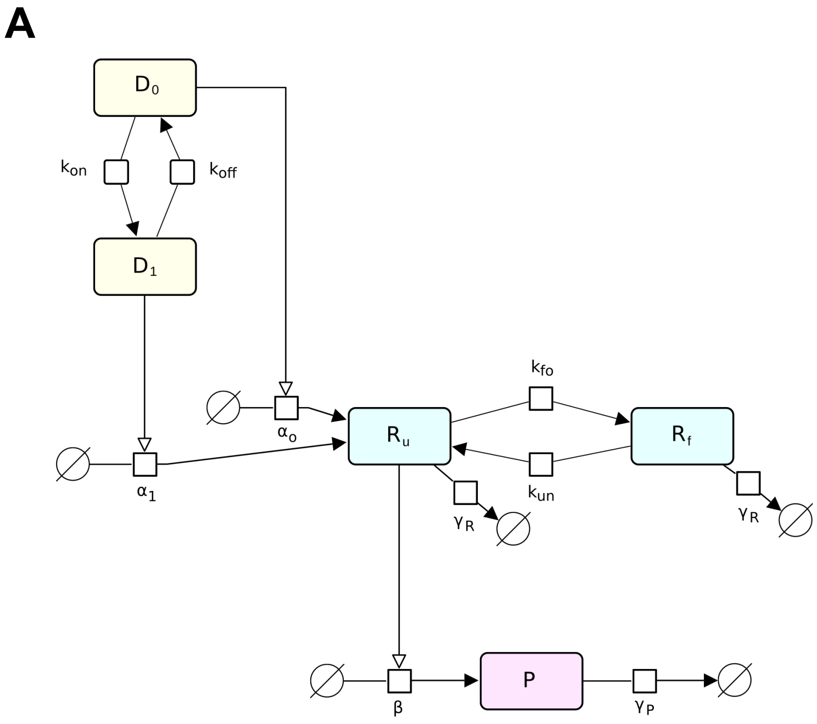

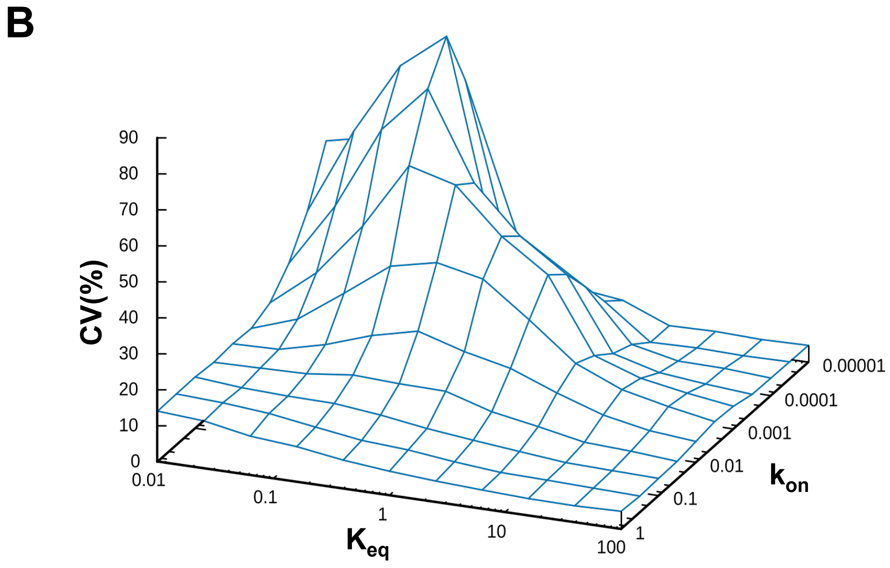

Figure S6. (*A*) A scheme showing reversible stochastic events in the gene expression pathway, featuring promoter on (D_1) and off (D_0) states) and mRNA (5'UTR) folded (R_f) and unfolded (R_u) states. P is protein, and γ_R and γ_P are degradation rates for mRNA and protein, respectively. (*B*) Predicted dependence of protein level noise as a function of kinetic parameters of *transcription*. This is presented for comparison with Fig. 7B.
